# Improving effective coverage of medical-oxygen services for neonates and children in health facilities in Uganda: a before–after intervention study

**DOI:** 10.1016/S2214-109X(24)00268-7

**Published:** 2024-08-14

**Authors:** Hamish R Graham, Freddy Eric Kitutu, Yewande Kamuntu, Blasio Kunihira, Santa Engol, Jasmine Miller, Absolom Zisanhi, Dorcas Kemigisha, Lorraine Nabbanja Kabunga, Charles Olaro, Harriet Ajilong, Freddie Ssengooba, Felix Lam

**Affiliations:** a Melbourne Children's Global Health, Murdoch Children's Research Institute, University of Melbourne, Royal Children's Hospital, Parkville, VIC, Australia; b Department of Paediatrics, University College Hospital, Ibadan, Nigeria; c Department of Pharmacy and Sustainable Pharmaceutical Systems Unit, School of Health Sciences, Makerere University, Kampala, Uganda; d Department of Health Policy Planning and Management, School of Health Sciences, Makerere University, Kampala, Uganda; e Clinton Health Access Initiative Uganda, Kampala, Uganda; f Clinton Health Access Initiative, Boston, MA, USA; g Directorate of Curative Services, Ministry of Health, Kampala, Uganda; h Uganda Paediatric Association, Kampala, Uganda; i Department of Women's and Children's Health, International Maternal and Child Health, Uppsala University, Uppsala, Sweden

## Abstract

**Background:**

Medical oxygen services are essential for the care of acutely unwell patients. We aimed to assess the effects of a multilevel, multicomponent health-system intervention on hypoxaemia detection, oxygen therapy, and mortality among neonates and children attending level IV health centres and hospitals in Uganda.

**Methods:**

For this before–after intervention study, we included children who attended paediatric or neonatal wards of 24 level IV health centres and seven general or regional referral hospitals in the Busoga and North Buganda regions of Uganda between June 1, 2020, and June 30, 2022. All neonates younger than 1 month and children aged 1 month to 14 years were eligible for inclusion. We excluded neonates who were not sick but stayed in the maternity ward for routine postnatal care. The intervention involved clinical training, mentorship, and supportive supervision; provision of pulse oximeters and cylinder-based oxygen sources; biomedical-capacity support; and support to develop and disseminate oxygen supply strategies, oxygen therapy guidelines, and lists of essential oxygen supplies. Trained research assistants extracted individual patient data from case notes using a standardised electronic data collection form. Data were collected on health-facility details, age, sex, clinical signs and symptoms, admission diagnoses, pulse oximetry readings, oxygen therapy details, and final patient outcome. The primary outcome was the proportion of admitted neonates and children with a pulse oximetry oxygen saturation reading documented in their patient case notes on day 1 of health-facility admission (ie, pulse oximetry coverage). We used mixed-effects logistic regression to evaluate the effect of the intervention.

**Findings:**

We obtained data on 71 997 eligible neonates and children admitted to 31 participating health facilities; the primary analysis included 10 001 patients in the pre-intervention period (ie, June 1 to Oct 30, 2020) and 51 329 patients in the post-intervention period (ie, March 1, 2021, to June 30, 2022). Because 1356 patients had missing data for sex, 4365 (46·7%) of 9347 in the pre-intervention group and 22 831 (46·2%) of 49 410 in the post-intervention group were female; 4982 (53·3%) in the pre-intervention group and 26 579 (53·8%) in the post-intervention group were male. The proportion of neonates and children with pulse oximetry at admission increased from 2365 (23·7%) of 10 001 in the pre-intervention period to 45 029 (87·7%) of 51 328 in the post-intervention period. Adjusted analysis indicated greater likelihood of a patient receiving pulse oximetry during the post-intervention period compared with the pre-intervention period (adjusted odds ratio 40·10, 95% CI 37·38–42·93; p<0·0001).

**Interpretation:**

Large-scale improvements in hospital oxygen services are achievable and have the potential to improve clinical outcomes. Governments should be encouraged to develop national oxygen plans and focus investment on interventions that have been shown to be effective, including the introduction of pulse oximetry into routine hospital care and clinical and biomedical mentoring and support.

**Funding:**

Bill & Melinda Gates Foundation and ELMA Philanthropies.

**Translations:**

For the Luganda and Lusoga translations of the abstract see Supplementary Materials section.

## Introduction

Medical oxygen is essential for the care of patients of all ages with acute, chronic, or surgical conditions.^1^, ^2^ However, many patients do not receive oxygen therapy due to under-recognition of hypoxaemia, low health-workforce competency, unreliable oxygen supply systems, and high cost to providers or patients.^3^ Improving access to medical oxygen requires addressing weaknesses at multiple levels and in multiple dimensions of medical oxygen systems, some of which were exacerbated by the COVID-19 pandemic and emphasised by *The Lancet Global Health* Commission on Medical Oxygen Security.^4^, ^5^

Multilevel engagement with health facility leadership, ward managers, individual health-care workers, and biomedical engineers and multicomponent support, such as provision of essential equipment, education, and quality improvement strategies, are prerequisites to successful implementation and sustainability of pulse-oximetry and oxygen-therapy solutions.^6^, ^7^, ^8^, ^9^, ^10^, ^11^ These interventions are collectively effective and have the potential to reduce risk of death from pneumonia by 50% and reduce all-cause mortality by 25% among children admitted to hospital in low-resource settings.^6^ However, little data are available on how to strengthen medical oxygen systems at scale and there are no data from low-income countries, such as Uganda.

**Research in context**

**Evidence before this study**

Medical oxygen services are essential for the care of patients of all ages with acute or chronic respiratory diseases, severe infections, trauma, neonatal conditions, obstetric complications, or conditions requiring surgery or anaesthesia. The COVID-19 pandemic highlighted existing gaps in the coverage of oxygen services globally and current evidence regarding how to improve oxygen-service coverage effectively, efficiently, and sustainably is largely limited to small-scale and medium-scale hospital-quality improvement programmes. To identify relevant evidence, we evaluated a systematic review of interventions to improve oxygen services for children and a systematic review of the cost-effectiveness of oxygen-systems strengthening. We then searched PubMed for interventional articles published between Jan 1, 2017, and Dec 15, 2023, in any language using the search terms “oxygen” AND “effectiveness”, excluding studies related to long-term oxygen therapy for chronic respiratory conditions. A study from Malawi reported that a large-scale health-systems intervention improved pneumonia management and led to a reduction in child pneumonia mortality. However, oxygen-related activities were a late inclusion to this programme and the study quality was low. Four hospital-based oxygen-quality improvement programmes reported reductions in pneumonia mortality among children admitted to hospital in Papua New Guinea, Laos, and Nigeria. Implementation of a solar-power system for lighting with low-energy lights and electricity to power two oxygen concentrators on a paediatric ward in a single-site study in Sierra Leone led to a reduction in mean in-patient mortality after a 6-month implementation period. A meta-analysis of these studies reported improved oxygen services for children younger than 5 years with pneumonia and overall. Although other studies have investigated specific aspects of oxygen services and potential innovations (eg, solar power, oxygen storage, or hub-and-spoke distribution models), we identified no large-scale studies addressing gaps in oxygen services.

**Added value of this study**

We evaluated a multilevel, multicomponent health-system intervention to improve medical oxygen-service coverage in Uganda. This intervention applied successful processes from hospital-based studies to a large-scale health-systems intervention at national, subnational, district, and facility levels. Led jointly by the Ugandan Ministry of Health and Clinton Health Access Initiative, the intervention was based on the National Scale Up of Medical Oxygen Implementation Plan 2018–2022 and included capacity building for health-care workers and biomedical technicians, catalytic procurement and repair of oxygen-related equipment, strengthening oxygen-logistic and supply-chain systems, and technical support for coordination of government and non-government stakeholders. We found an increase in pulse-oximetry coverage and oxygen-service coverage for neonates and children with hypoxaemia during the post-intervention period. Improvements were greater in smaller, low-level health facilities than in larger, high-level hospitals.

**Implications of all the available evidence**

Our findings suggest that large-scale improvements in hospital oxygen services in African countries are achievable and have the potential to improve clinical outcomes. Development and implementation of context-sensitive national oxygen plans could be an effective way for governments and global health agencies to scale-up and sustain medical-oxygen services globally.

Uganda is a low-income country with a gross national income per capita of US$974 in 2022.^12^ Although mortality in children younger than 5 years is estimated to be 52 deaths per 1000 livebirths, infant mortality is estimated to be 36 deaths per 1000 livebirths, neonatal mortality is estimated to be 22 deaths per 1000 livebirths, and pregnancy-related mortality ratio is estimated to be 228 deaths per 100 000 livebirths,^13^, ^14^ the leading causes of hospital admission are malaria, pneumonia, anaemia, sepsis, neonatal conditions, road-traffic accidents or other injuries, complications of pregnancy or obstructed labour, sickle cell disease, and asthma^12^ associated with hypoxaemia, for which pulse oximetry and oxygen therapy are the basic standard of care.^1^, ^2^

Uganda has a tiered health-care structure and a decentralised governance structure; some responsibilities, such as the health-commodity supply chain and infrastructural and biomedical equipment plans, are managed centrally by the Ministry of Health and its agencies, including the National Medical Stores and the National Drug Authority. Health facilities provide services according to their level in the health system (appendix 3 p 2).^15^, ^16^ In 2018, the Ugandan Ministry of Health and Clinton Health Access Initiative (CHAI), in consultation with National Medical Stores, WHO country office, UNICEF, Joint Medical Store, Makerere University, Uganda Peadiatric Association, national and regional referral hospitals, and district-level local governments, developed the National Scale Up of Medical Oxygen Implementation Plan 2018–2022^17^ as a strategic blueprint to strengthen medical-oxygen services across Uganda.

We aimed to assess the effects of a multilevel, multicomponent health-system intervention based on the National Scale Up of Medical Oxygen Implementation Plan 2018–2022 on hypoxaemia detection, oxygen therapy, and mortality among neonates and children attending level IV health centres and hospitals in Uganda.

## Methods

### Study design

For this before–after intervention study, we included children who attended paediatric or neonatal wards of 24 level IV health centres and seven general or regional referral hospitals in the Busoga and North Buganda regions of Uganda between June 1, 2020, and June 30, 2022 (appendix 3 p 6).

This study received ethical approval from the Research Ethics Committee at Makerere University (#SHSREC reference number 2020–030) and received clearance and registration from the Uganda National Council of Science and Technology (reference number HS631ES).

### Patients

We chose the North Buganda and Busoga regions because of their higher infant and child mortality rates compared with other regions in Uganda in 2022–23.^18^ We included all government general hospitals and level IV health centres in the catchment areas of the two regional referral hospitals where inpatient services for neonates and children are provided. Level IV health centres are a tier lower than general hospitals and operate at the county level; they serve a 100 000 target population with in-patient capacity of 24 beds and 50 staff. They provide preventive, promotive, outpatient, curative, maternity, in-patient, emergency, and minor life-saving surgeries including ceaserean section. They also provide blood-transfusion, mortuary, laboratory, and ultrasound examination services.

All neonates younger than 1 month and children aged 1 month to 14 years were eligible for inclusion. We excluded neonates who were not sick but stayed in the maternity ward for routine postnatal care. This study did not include a control group.

Makerere University School of Health Sciences Research and Ethics Committee approved a waiver of informed consent for use of routinely collected clinical data.

### Intervention

On the basis of previous work in Uganda and Nigeria^19^, ^20^ and stakeholder consultation, we developed a multilevel, multicomponent health-system intervention to be used within Uganda's existing medical-oxygen system (appendix 3 p 2).

At the national level, the Ugandan Ministry of Health revised national medical oxygen equipment and devices specifications, procurement guidelines, hypoxaemia management guidelines, the essential medicine and health supplies list for oxygen therapy, and strategies to strengthen biomedical engineering capacity, workshops, and processes and spare-part supply systems. At the health-facility level, health-facility managers were trained in oxygen-related health-worker capacity building;, training and mentorship of clinical, supply-chain, and biomedical-engineering staff, enhanced equipment inventory and distribution of medical oxygen, and documentation of pulse oximetry use and oxygen therapy in routine patient records.

In regional referral hospitals, pressure-swing-adsorption oxygen generators were refurbished. Their piping systems and regional biomedical workshops were equipped with spare-parts kits (ie, circuit breakers, printed circuit boards, sieve beds, compressor components, valves, wheels, motor capacitors, inlet filters, and outlet bacteria filters and analysers), as specified by the manufacturer. In health facilities, staff training, clinical mentorship, oxygen champions (ie, health workers selected from health-facility staff to participate in the clinical mentoring and on-job training component of the intervention), and clinical audit and feedback were implemented (appendix 3 p 2). The Ministry of Health and CHAI provided pulse oximeters, oxygen concentrators, oxygen cylinders, and related accessories, including regulators, spanners, flow splitters, and nasal prongs, to all included level IV health centres and hospitals by Jan 30, 2021, which enabled health facilities to provide basic low-flow oxygen-therapy services to paediatric patients.

Components across multiple levels of health care were implemented or enhanced, such as supply chains, distribution of oxygen to low-level health facilities through a regional hub-and-spoke model (ie, a distribution system in which medical oxygen is picked from a central or regional oxygen source and distributed to multiple service-delivery points at health centres),^21^ a new equipment inventory database and tracking system involving Ugandan National Medical Stores in mapping, linking oxygen suppliers with health facilities, advocating for recruitment of more biomedical personnel in regional referral hospitals and general hospitals, and revitalising regional biomedical workshops.

### Procedures

To maximise data completeness and ensure data quality, we used a revised patient register form to capture summary data on oxygen saturation (SpO_2_) and oxygen therapy. This revised patient register form was integrated into the standard health-management information system reporting to the Ugandan Ministry of Health. The revised patient-register form was discussed and ratified by the Ugandan Ministry of Health, pilot-tested in two Regional referral hospitals in 1382 patients in Jinja and Mubende during 3 months and revised with input from the national team (YK, BK, SE, AZ, DK, LNK, CO, and HA) and academic partners (HRG and FEK).

Trained research assistants extracted individual patient data from case notes using a standardised electronic data collection form on SurveyCTO between June 1, 2020, and June 30, 2022; the start was delayed by 5 months due to COVID-19 restrictions. They checked ward registers to ensure the inclusion of all eligible patients. Alongside regular supportive supervision visits by clinical mentors and supervisors (FEK, SE, and HA), led by members of the study district health offices in Uganda and national quality-improvement programmes,^22^ BK and JM checked the quality of patient documentation in patient registers and charts and reviewed the quality of data extraction. Data were collected on health facility details, age, sex, clinical signs and symptoms, admission diagnoses, pulse oximetry readings, oxygen therapy details, and final patient outcome. Sex data were self-reported by participants or from direct observation by a health worker. The options of male or female were provided.

Phase 1 training in health facilities that acquired medical oxygen equipment and source earlier occurred between Oct 26 and Oct 30, 2020. Phase 2 training of the remaining health facilities occurred between Feb 22 and Feb 26, 2021. A training and clinical-mentorship package was delivered in a 5-day workshop offsite, followed by 3 days of on-job support supervision and clinical mentorship at the health facilities. The competencies intended for the health-care workers included identifying danger signs; triaging sick children; using pulse oximetry for timely hypoxaemia detection; risk stratification; and monitoring of prognosis and oxygen administration including oxygen-delivery techniques, proper handling and preventive maintenance of oxygen equipment, and supply-chain management. There was emphasis on accurate patient data documentation, analysis, and use for continuous quality improvement for oxygen therapy. The training methods used were of didactic and participatory teaching sessions, including group discussion, one-on-one discussions, case scenarios, demonstrations, simulation and practice on oxygen equipment, and patient assessment and clinical practice at the nearby hospital applying adult learning principles. 2-day clinical mentorship visits for level IV health facilities and 3-day clinical mentorship visits for general and regional referral hospitals were conducted in the last week of every second month between Nov 1, 2020, and Nov 30, 2021.

The primary outcome was the proportion of admitted neonates and children with a pulse oximetry SpO_2_ reading documented in their patient case notes on day 1 of hospital admission (ie, pulse oximetry coverage). Secondary outcomes were the proportion of neonates and children admitted to hospital with hypoxaemia who received oxygen on admission (ie, oxygen service coverage), the proportion of admitted neonates and children who received oxygen on hospital admission (ie, oxygen use), the proportion of patients receiving oxygen on hospital admission who had hypoxaemia (ie, appropriate oxygen use), and the proportion of neonates and children who died in hospital (ie, all-cause mortality).

Selection bias was minimised by taking universal samples of all eligible patients attending the health facilities during the study period. Furthermore, the data-collection tools were pilot-tested and adapted with input from health-care workers at the study health facilities before use and both health-care workers and research assistants were trained on the study protocol, intervention components, and data collection by the study team (FEK, YK, BK, SE, AZ, DK, and HA). Routine data-duality checks were done by BK and JM and feedback was provided to the research assistants.

### Statistical analysis

From an implementation-science perspective, we aimed to maximise the number of patients eligible for the primary and secondary outcomes by using a universal sample. A priori, we estimated more than 99% power to detect a change in the pulse oximetry coverage, the primary outcome, from 10% to 75%. Assuming 7·5% hypoxaemia prevalence,^23^ we estimated 93% power to detect a change in the secondary outcome of oxygen-service coverage to patients with hypoxaemia (intracluster correlation coefficient [ICC] 0·05) from 50% to 60%. We considered the 31 study health facilities as clusters and assumed an ICC of 0·5^10^ to account for homogeneity of observations within a health facility. At the end of the study, the achieved sample size had 99% power to detect hypothesised change in pulse oximetry coverage and 86% power to detect hypothesised change on oxygen-service coverage.

We presented study outcomes descriptively by population group (ie, neonatal admissions and post-neonatal admissions) and health facility (ie, level IV health centre, general hospital, regional referral hospital, and overall), summarised continuous data using mean (SD) or median (IQR) as appropriate, and summarised categorical data using proportions.

We conducted the primary effectiveness analysis by comparing pulse oximetry coverage between baseline and the post-intervention period, with a 4-month washout period for the intervention to have an effect (ie, 10-month pre-intervention period and 18-month post-intervention period). This short washout period was based on previous data indicating that pulse oximetry and oxygen practices take 3–6 months to become routine.^24^ The pre-intervention period was from June 1 to Oct 30, 2020, and the post-intervention period was from March 1, 2021, to June 30, 2022.

We used mixed-effects logistic regression to evaluate the effect of the intervention in regard to the primary and secondary outcomes using random effects to adjust for clustering at individual health facilities and fixed effects to adjust for age (ie, child *vs* neonate) and sex (ie, male *vs* female). We extended this mixed-effects model to explore individual and contextual influences on clinical outcomes by adding additional fixed effects for health-facility type and clinical signs or symptoms on admission. The primary analysis model was adjusted for age, sex, and facility. The extended analysis model was adjusted for age, sex, facility, region, health facility, cough, fast or difficult breathing, chest indrawing, convulsions, altered conscious state, fever, ability to feed or drink, and severe respiratory distress.

We only included participants for whom we had no missing data on the variables of interest for the respective analyses.

We reported hypoxaemia prevalence, likelihood of death, and summary statistics on oxygen-therapy duration and flow rates (as recorded by health-care workers and trained research assistants) with analysis restricted to the final 12 months of the post-intervention period to best reflect post-intervention care practices in the context of routine pulse oximetry. We conducted exploratory analysis of these post-intervention data to explore why some patients with documented hypoxaemia on admission did not receive oxygen. We estimated numbers of additional patients who received pulse oximetry and oxygen during the intervention period and lives saved using effect estimates from this study and a previous meta-analysis.^6^

We used Stata version 18 for all analyses. We report this study and our findings according to the revised Standards for Quality Improvement Reporting Excellence.^25^

### Role of the funding source

The funders of the study had no role in study design, data collection, data analysis, data interpretation, or writing of the report.

## Results

We obtained data on 71 997 eligible neonates and children admitted to 31 participating health facilities between June 1, 2020, and June 30, 2022 (appendix 3 p 6). After excluding 10 667 admitted during the 4-month washout period, the primary analysis included 10 001 patients in the pre-intervention period (ie, June 1 to Oct 30, 2020) and 51 329 patients in the post-intervention period (ie, March 1, 2021, to June 30, 2022; table 1). Because 1356 patients had missing data for sex, 4365 (46·7%) of 9347 in the pre-intervention group and 22 831 (46·2%) of 49 410 in the post-intervention group were female; 4982 (53·3%) in the pre-intervention group and 26 579 (53·8%) in the post-intervention group were male. The post-intervention population included fewer neonates, older median age, and more neonates and children admitted to level IV health centres (table 1). Neonatal encephalopathy, neonatal sepsis, and preterm birth accounted for more than 90% of neonatal admission diagnoses; malaria, diarrhoeal disease, pneumonia, and sepsis accounted for more than 90% of post-neonatal admission diagnoses (table 1). We observed an increase in monthly admissions over time, particularly in level IV health centres (appendix 3 p 7).

**Table 1:** Sociodemographic and clinical characteristics of patients

		**Pre-intervention group (June to October, 2020; n=10 001)**	**Post-intervention group (March, 2021, to June, 2022; n=51 329)**	**p value**
Care attended		..	..	<0·0001
	Level IV health centre	2276 (22·8%)	20 897 (40·7%)	..
	General hospital	4507 (45·1%)	18 935 (36·9%)	..
	Regional referral hospital	3218 (32·2%)	11 497 (22·4%)	..
Age*		..	..	<0·0001
	Neonates (aged <1 month)	3177 (31·8%)	12 393 (24·1%)	..
	Infants (aged 1–11 months)	1041 (10·4%)	7164 (14·0%)	..
	Young child (aged 12–59 months)	3073 (30·7%)	20 058 (39·1%)	..
	Older child (aged 5–9 years)	1214 (12·1%)	8208 (16·0%)	..
	Young adolescent (aged 10–14 years)	461 (4·6%)	2787 (5·4%)	..
Median age, months		13 (0–42)	18 (1–48)	<0·0001
Sex*		..	..	0·38
	Female	4365/9347 (46·7%)	22 831/49 410 (46·2%)	..
	Male	4982/9347 (53·3%)	26 579/49 410 (53·8%)	..
Region		..	..	<0·0001
	North Buganda	3274 (32·7%)	15 517 (30·2%)	..
	Busoga	6727 (67·3%)	35 812 (69·8%)	..
Presenting signs and symptoms†				
	Fever, chills, or shivering	5854 (58·5%)	34 009 (66·3%)	<0·0001
	Cough	2369 (23·7%)	19 992 (39·0%)	<0·0001
	Fast or difficult breathing	1452 (14·5%)	10 283 (20·0%)	<0·0001
	Chest indrawing	161 (1·6%)	813 (1·6%)	0·85
	Severe respiratory distress (ie, cyanosis, grunting, nasal flaring, or stridor)	520 (5·2%)	3194 (6·2%)	<0·0001
	Other respiratory symptoms (eg, wheezing, noisy, or slow)	323 (3·2%)	1948 (3·8%)	0·0062
	Diarrhoea	1228 (12·3%)	8629 (16·8%)	<0·0001
	Altered conscious state (eg, drowsy or unconscious)	933 (9·3%)	3767 (7·3%)	<0·0001
	Seizures	728 (7·3%)	3817 (7·4%)	0·58
	Pain (eg, abdominal, other, or infant grimace)	1594 (15·9%)	11 925 (23·2%)	<0·0001
	Poor feeding or drinking (eg, vomiting)	3243 (32·4%)	17 146 (33·4%)	0·058
Mean heart rate, beats per minute		124 (26)	122 (26)	<0·0001
Mean respiratory rate, breaths per minute		39·5 (15·8)	37·7 (13·4)	<0·0001
Signs of hypoxaemia (eg, respiratory distress, chest indrawing, cyanosis, grunting, nasal flaring, drowsy, or unconscious)		730 (7·3%)	3930 (7·7%)	0·22
WHO emergency signs (eg, altered consciousness, severe respiratory distress, unable to feed or drink, or chest indrawing in older child)		4260 (42·6%)	21 541 (42·0%)	0·24
Severe hypoxaemia (ie, oxygen saturation <90%)		518/2365 (21·9%)	5450/45 029 (12·1%)	<0·0001
Moderate hypoxaemia (ie, oxygen saturation 90–93%)		369/2365 (15·6%)	4486/45 029 (10·0%)	<0·0001
Neonatal admission diagnoses‡				
	Neonatal encephalopathy	1073/3177 (33·8%)	4195/12 393 (33·9%)	0·94
	Neonatal sepsis	879/3177 (27·7%)	4135/12 393 (33·4%)	<0·0001
	Small or preterm	913/3177 (28·7%)	4033/12 393 (32·5%)	<0·0001
	Jaundice	107/3177 (3·4%)	575/12 393 (4·6%)	0·0018
Post-neonatal admission diagnoses‡				
	Malaria	4264/6824 (62·5%)	22 764/38 936 (58·5%)	<0·0001
	Pneumonia	687/6824 (10·1%)	5865/38 936 (15·1%)	<0·0001
	Diarrhoeal disease	1211/6824 (17·8%)	8600/38 936 (22·1%)	<0·0001
	Sepsis	1016/6824 (14·9%)	6148/38 936 (15·8%)	0·059
	WHO pneumonia	361/6824 (5·3%)	3471/38 936 (8·9%)	<0·0001
	WHO severe pneumonia	186/6824 (2·7%)	1879/38 936 (4·8%)	<0·0001

Data are n (%), n/N (%), median (IQR), or mean (SD). p values were calculated with Pearson χ^2^, Student's *t* test, or Stata χ^2^ median command, as appropriate. WHO pneumonia and severe pneumonia were classified on the basis of documentation of clinical signs: cough and fast or difficult breathing with or without severe respiratory distress or other emergency signs (eg, hypoxaemia, altered conscious state, seizures, or unable to feed or drink).

* Some patients had missing age data or missing sex data.

† Some patients presented with more than one sign or symptom.

‡ Some patients had more than one diagnosis.

**Figure 1:**
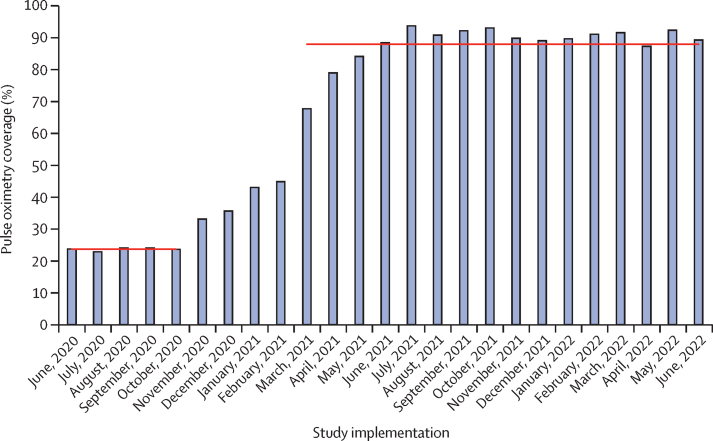
Pulse oximetry coverage over time Pulse oximetry coverage was defined as the proportion of patients admitted to hospital with documented oxygen saturation on day 1 of admission. Horizontal lines indicate pre-intervention and post-intervention means.

The proportion of neonates and children with pulse oximetry at admission increased from 2365 (23·7%) of 10 001 in the pre-intervention period to 45 029 (87·7%) of 51 328 in the post-intervention period (figure 1; table 2). Improvement reached the full coverage target of 80% in approximately 6 months and was maintained during 18-month follow-up for all levels of health care, regardless of baseline coverage (appendix 3 p 15).

**Table 2:** Effect of strengthening activities for oxygen systems on pulse-oximetry practices, oxygen practices, and in-hospital mortality

	**Number of patients**	**Mixed-model adjusted odds ratio (95% CI)**		**Intracluster correlation coefficient (95% CI)**	**p value**
		Primary-analysis model	Extended-analysis model		
**Pulse oximetry coverage (primary outcome)**					
Pre-intervention	2365/10 001 (23·7%)	..	..	..	..
Post-intervention	45 029/51 329 (87·7%)	40·10 (37·38–42·93)	39·20 (36·55–42·05)	0·26 (0·17–0·37)	<0·0001
**Oxygen service coverage (secondary outcome)**					
Pre-intervention	358/899 (39·8%)	..	..	..	..
Post-intervention	4074/5708 (71·4%)	3·81 (3·26–4·46)	3·90 (3·32–4·59)	0·09 (0·05–0·16)	<0·0001
**Oxygen use (secondary outcome)**					
Pre-intervention	788/10 001 (7·9%)	..	..	..	..
Post-intervention	5789/51 329 (11·3%)	1·95 (1·79–2·13)	1·73 (0·57–1·91)	0·12 (0·07–0·18)	<0·0001
**Appropriate use of oxygen (secondary outcome)**					
Pre-intervention	358/788 (45·4%)	..	..	..	..
Post-intervention	4074/5789 (70·4%)	3·18 (2·69–3·75)	2·91 (2·45–3·44)	0·20 (0·12–0·32)	<0·0001

Data are n/N (%), unless otherwise stated. The primary-analysis model was adjusted for age, sex, and facility. The extended-analysis model was adjusted for age, sex, region, health facility, cough, fast or difficult breathing, chest indrawing, convulsions, altered conscious state, fever, ability to feed or drink, and severe respiratory distress. Pulse oximetry coverage was defined as the proportion of patients admitted to hospital with documented SpO_2_ on day 1 of admission. Oxygen service coverage was defined as the proportion of patients with hypoxaemia (ie, SpO_2_ <90% or clinical signs of hypoxaemia if pulse oximetry not done) who received oxygen on day 1 of hospital admission. Oxygen use was defined as the proportion of patients who received oxygen on day 1 of hospital admission. Appropriate use of oxygen was defined as the proportion of patients receiving oxygen who had SpO_2_ <90% or clinical signs of hypoxaemia (if oximetry not done). Severe hypoxaemia was defined as SpO_2_ <90%, as per WHO guidelines, or presence of clinical signs of hypoxaemia if SpO_2_ was not documented.^23^ Although this definition might miss some patients who needed oxygen therapy without having severe hypoxaemia (eg, initial resuscitation of patients who are moribund or patients with moderate hypoxaemia and additional risk factors, such as severe anaemia, heart failure, or head injury),^23^, ^24^ it enabled consistent reporting under WHO guidelines and other oxygen-access studies.^9^, ^10^, ^20^ SpO_2_=oxygen saturation.

Adjusted analysis indicated greater likelihood of a patient receiving pulse oximetry during the post-intervention period compared with the pre-intervention period (adjusted odds ratio [aOR] 40·10, 95% CI 37·38–42·93; p<0·0001; table 2; appendix 3 p 11). In primary adjusted analysis, pulse oximetry was less likely to be done for children (aged 1 month to 14 years) than for neonates (aged <1 month; 0·44, 0·41–0·47; p<0·0001) and less likely to be done for male patients than for female patients (0·94, 0·90–0·99; p=0·026). Extended analysis results were similar (table 2). We estimated that an additional 10 810 neonates and 33 258 children had pulse oximetry during the 16-month post-intervention period than would have been expected without the intervention (appendix 3 p 13).

**Figure 2:**
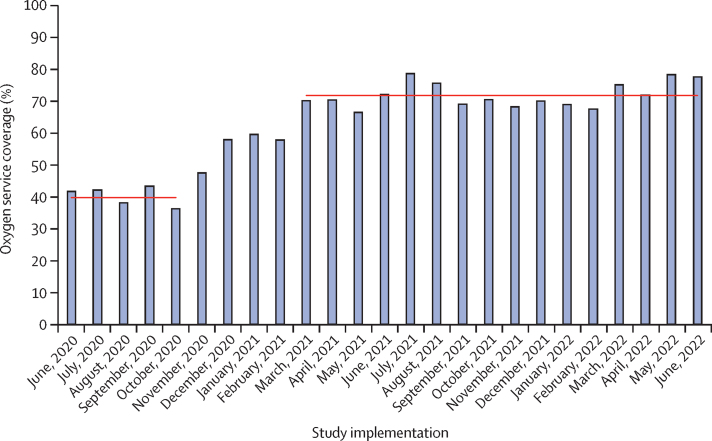
Oxygen service coverage over time Oxygen service coverage was defined as the proportion of patients with hypoxaemia (ie, oxygen saturation <90% or clinical signs of hypoxaemia if pulse oximetry not done) who received oxygen on day 1 of hospital admission. Horizontal lines indicate pre-intervention and post-intervention means.

Oxygen service coverage for neonates and children with hypoxaemia (ie, SpO_2_<90% or signs of hypoxaemia if SpO_2_ not documented) increased from 358 (39·8%) of 899 patients pre-intervention to 4074 (71·4%) of 5708 patients post-intervention (figure 2; table 2). This change occurred incrementally during 6 months and was maintained during 18-month follow-up for all levels of health care, regardless of baseline coverage (appendix 3 p 15).

Adjusted analysis indicated greater likelihood of oxygen provision to children with hypoxaemia during the post-intervention period than during the pre-intervention period (aOR 3·81, 95% CI 3·26 to 4·46; p<0·0001). Oxygen was less likely to be given to children with hypoxaemia than neonates with hypoxaemia (0·56, 0·50–0·63; p<0·0001) with no sex difference (1·02, 0·91–1·14; p=0·79).

**Figure 3:**
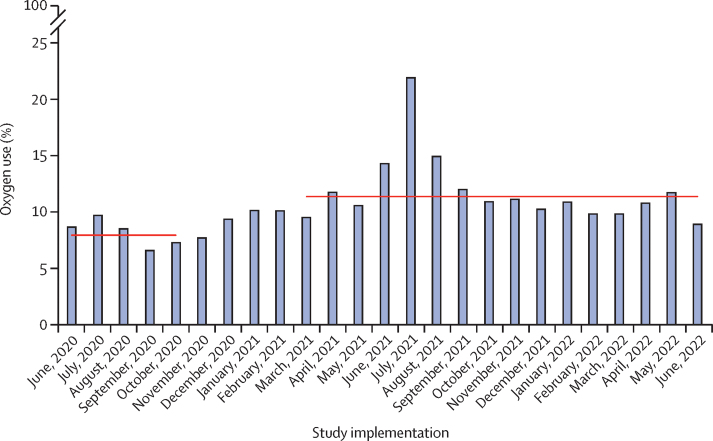
Oxygen use over time Oxygen use was defined as the proportion of patients who received oxygen on day 1 of hospital admission. Horizontal lines indicate pre-intervention and post-intervention means.

Oxygen use increased from 788 (7·9%) of 10 001 patients pre-intervention to 5789 (11·3%) of 51 329 patients post-intervention, with a larger proportional change among children (from 587 [18·5%] of 3177 to 3556 [28·7%] of 12 393 for neonates *vs* from 201 [3·0%] of 6824 to 2233 [5·7%] of 38 936 for children) but a greater-percentage point change among neonates (neonates 10·2 percentage points *vs* children 2·8 percentage points; figure 3). Oxygen use was higher for neonates and children in regional referral hospitals (1178 [33·6%] of 3507 neonates and 920 [11·5%] of 7990 children during the post-intervention period) than in general hospitals (1612 [26·1%] of 6180 neonates and 583 [4·6%] of 12 755 children during the post-intervention period) and level IV health centres (766 [28·3%] of 2706 neonates and 730 [4·0%] of 18 191 children during the post-intervention period; appendix 3 p 17). We estimated that an additional 1623 neonates and 1266 children received oxygen during the 16-month post-intervention period than would have been expected without the intervention (appendix 3 p 13).

During the pre-intervention period, less than half of patients who received oxygen on admission had documented hypoxaemia, increasing to two-thirds in the post-intervention period (appendix 3 p 11). Adjusted analysis indicated that neonates and children were more likely to be given appropriate oxygen during the post-intervention period than during the pre-intervention period (aOR 3·18, 95% CI 2·69–3·75; p<0·0001; table 2; appendix 3 p 11).

Exploratory analysis of the final 12 months post-intervention suggested that use of oxygen was largely rational, guided by degree of hypoxaemia and presence of clinical signs of hypoxaemia (appendix 3 p 14). However, 568 (23·0%) of 2469 neonates and 489 (30·4%) of 1608 children with hypoxaemia at hospital admission did not promptly receive oxygen therapy. Of these patients, 205 (36·1%) of 568 neonates and 41 (8·4%) of 489 children had signs of hypoxaemia, 352 (62·0%) neonates and 179 (36·6%) children had WHO emergency signs, and 99 (17·4%) neonates and 35 (7·2%) children died in hospital. However, 360 (63·4%) of 568 neonates and 174 (35·6%) of 489 children with hypoxaemia who did not receive oxygen immediately on admission did receive it after a delay (appendix 3 p 14), and few still had hypoxaemia on days 2 and 3 of admission.

During the final 12 months post-intervention, during which pulse oximetry coverage was more than 90% and oxygen therapy was more available than pre-intervention (figure 1), prevalence of severe hypoxaemia (ie, SpO_2_ <90%) was 4077 (11·5%) of 35 314 patients (2469 [28·4%] of 8702 neonates, 557 [11·3%] of 4913 infants aged 1–11 months, 784 [5·7%] of 13 696 children aged 1–4 years, and 195 [3·4%] of 5657 older children aged 5–14 years) and was associated with 12·82 (95% CI 11·28–14·56) likelihood of death compared with prevalence of moderate hypoxaemia (ie, SpO_2_ 90–93%; table 3). An additional 3325 (9·4%) of 35 314 patients had moderate hypoxaemia (1301 [15·0%] of 8702 neonates, 475 [9·7%] of 4913 infants, 1040 [7·6%] of 13 696 children, and 350 [6·2%] of 5657 older children) and was associated with 3·02 (2·41–3·75) likelihood of death compared with prevalence of severe hypoxaemia (table 3). Increased hypoxaemia prevalence rates were observed in younger children and patients attending general and regional referral hospitals (table 3). Neonates and children without documented SpO_2_ had high odds of death, more so than those with severe hypoxaemia, but low rates of oxygen prescription (even if they had signs of respiratory distress; appendix 3 p 14). Assuming similar prevalence of hypoxaemia during the pre-intervention period, we estimated that 511 (56·7%) of 902 neonates and 286 (69·2%) of 413 children with severe hypoxaemia were not detected during the 5-month pre-intervention period.

**Table 3:** Prevalence of moderate and severe hypoxaemia and likelihood of death by age and health facility from July, 2021, to June, 2022

		**Number of patients**	**Case fatality rate**	**Moderate hypoxaemia (ie, SpO_2_ 90–93%)**			**Severe hypoxaemia (ie, SpO_2_ <90%)**		
				Number of patients	Prevalence	Likelihood of death	Number of patients	Prevalence	Likelihood of death
Overall		35 314	3·20%	3325	9·42 (9·11–9·72)	3·02 (2·41–3·75)	4077	11·55 (11·21–11·88)	12·82 (11·28–14·56)
Age*									
	Neonates (aged <1 month)	8702	8·13%	1301	14·95 (14·21–15·72)	1·65 (1·20–2·24)	2469	28·37 (27·43–29·33)	6·59 (5·54–7·84)
	Infants (aged 1–11 months)	4913	2·69%	475	9·67 (8·86–10·53)	2·62 (1·35–4·80)	557	11·34 (10·46–12·26)	8·91 (6·12–12·97)
	Young child (aged 12–59 months)	13 696	1·31%	1040	7·59 (7·16–8·05)	4·20 (2·61–6·58)	784	5·72 (5·34–6·13)	11·31 (8·14–15·64)
	Older child (aged 5–9 years)	5657	1·38%	350	6·19 (5·57–6·85)	2·28 (0·78–5·46)	195	3·45 (2·99–3·96)	23·00 (13·65–38·22)
	Young adolescent (aged 10–14 years)	1833	1·85%	136	7·42 (6·26–8·72)	2·53 (0·62–7·77)	56	3·06 (2·32–3·95)	16·85 (6·71–39·35)
Level IV health centre		14 651	1·04%	1064	7·26 (6·85–7·69)	1·73 (0·66–3·85)	1135	7·75 (7·32–8·19)	20·70 (14·55–29·62)
	Neonates	1863	4·06%	272	14·60 (13·03–16·29)	0·27 (0·01–1·77)	592	31·78 (29·67–33·95)	8·24 (4·52–15·85)
	Children (aged 1 month to 14 years)	12 788	0·62%	792	6·19 (5·78–6·63)	2·54 (0·87–6·14)	543	4·25 (3·90–4·61)	20·40 (12·48–33·12)
General hospital		12 674	3·21%	1354	10·68 (10·15–11·22)	2·91 (2·04–4·09)	1490	11·76 (11·20–12·33)	10·81 (8·74–13·37)
	Neonates	4503	6·34%	632	14·04 (13·03–15·08)	1·29 (0·77–2·10)	1021	22·67 (21·46–23·93)	6·78 (5·21–8·83)
	Children (aged 1 month to 14 years)	8171	1·48%	722	8·84 (8·23–9·47)	5·77 (3·42–9·55)	469	5·74 (5·25–6·27)	11·07 (7·36–16·50)
Regional referral hospital		7989	7·02%	907	11·35 (10·67–12·07)	2·44 (1·77–3·34)	1452	18·17 (17·33–19·04)	8·69 (7·22–10·47)
	Neonates	2336	14·49%	397	16·99 (15·49–18·58)	2·05 (1·31–3·20)	856	36·64 (34·69–38·63)	5·64 (4·35–7·36)
	Children (aged 1 month to 14 years)	5653	3·92%	510	9·02 (8·29–9·80)	1·77 (1·03–2·91)	596	10·54 (9·75–11·37)	7·69 (5·73–10·29)

Data are n or odds ratio (95% CI), unless otherwise specified. SpO_2_=oxygen saturation.

* Some patients had missing age data.

Among neonates and children who received oxygen therapy, oxygen was provided for a median of 1 day (IQR 1–2) for neonates and a median of 1 day (IQR 1–2) for children, with median recorded flow rate of 1·0 L per min (IQR 0·5–1·0) for neonates and 2·0 L per min (IQR 1·0–2·0) for children.

During the pre-intervention period 224 (7·8%) of 2870 neonates and 120 (1·9%) of 6173 children died in hospital, compared with 976 (8·2%) of 11 878 neonates and 584 (1·6%) of 37 483 children during the post-intervention period. All-cause mortality in hospital was higher in neonates (aged <1 month) and infants (aged 1–11 months) than in older age groups (appendix 3 p 11). We estimated that 145 child deaths from pneumonia and 154 child deaths overall were averted during the 16-month post-intervention period than would have been expected pre-intervention (appendix 3 p 13).

## Discussion

Our study is the first evaluation of a multilevel, multicomponent health-system intervention based on the Ugandan National Scale Up of Medical Oxygen Implementation Plan 2018–2022.^17^ We found large and rapid improvements in pulse oximetry coverage and oxygen-service coverage among eligible neonates and children across health facilities, especially level IV health centres and general hospitals. Pulse oximetry coverage is a clinically relevant outcome that correlates highly with appropriate use of oxygen therapy and provides the best single indicator of oxygen access to patients.^19^

The approach to strengthening hospital oxygen systems by the Ugandan Ministry of Health and CHAI contained three elements described in previous successful programmes: health-care worker and biomedical-engineer capacity building and interprofessional teams, provision of pulse oximeters and essential oxygen-delivery equipment, and enhanced medical-oxygen supply.^20^ However, the way these elements were implemented was novel. First, Uganda was one of the first countries with a national oxygen scale-up plan that guided intervention implementation. Second, health-care worker capacity building was based on a clinical mentoring programme led by members of the study district health offices in Uganda and national quality-improvement programmes. Third, although previous programmes typically used oxygen concentrator-based supply and delivery systems, this programme focused on strengthening oxygen cylinder-distribution networks that depend on pressure swing adsorption oxygen generators located at regional referral hospitals for refill. Fourth, the intervention was implemented within routine Ministry of Health and CHAI systems, including national activities such as the development of normative guidance, national logistic-management systems, support for forecasting and budgeting, and conduct of health-facility surveys. Although this approach introduced complexity and challenges (eg, the hub-and-spoke distribution model as a strategy for sustainable supply), it could have supported long-term sustainability, efficiency, and scalability.

This study reports how a multilevel, multicomponent health-system intervention improved pulse oximetry and oxygen use at scale in real-world settings. However, we acknowledge the need to report other implementation outcomes including reach, appropriateness, implementation cost, and sustainability of this and future work.

Improvements in pulse oximetry and oxygen service coverage were greater in smaller, low-level health facilities than in larger, high-level hospitals, which had lower baseline equipment availability and lower clinical and biomedical staff capacity. Notably, low-level health facilities and hospitals are where a large proportion of child admissions and hospital deaths occur.^18^ Level IV health centres and general hospitals provided care for approximately 70% of neonates and children in this study, including 64% of those with hypoxaemia. Many responses to the global oxygen crisis focus on large facilities, with components such as liquid oxygen, onsite oxygen generators at large hospitals, and increased end-ventilatory equipment.^26^ Future efforts to improve oxygen systems should include low-level health facilities that provide care to populations who have low income or are difficult to reach, for increased equity and spread of medical oxygen-therapy benefits.

Our results are consistent with previous studies regarding use of pulse oximetry to identify critically ill patients, detect hypoxaemia, and guide oxygen therapy.^10^, ^23^, ^27^ Hypoxaemia is both common and one of the strongest risk factors for death for neonates and children admitted to hospital in low-income and middle-income countries.^23^, ^28^, ^29^ Our findings of rapid implementation of pulse oximetry and improved oxygen use are similar to findings from Nigeria, where pulse oximetry oxygen therapy use led to a reduction in mortality in children with acute lower respiratory infection.^10^, ^27^ Universal access to pulse oximetry should be a priority for the care of acutely unwell neonates and children.

Our study provides insights into some of the quality gaps in oxygen therapy in Uganda that have been reported in previous literature.^7^, ^9^, ^10^, ^11^, ^27^, ^30^ Not all patients with documented hypoxaemia were given oxygen on admission, even after oxygen supplies were reported to be reliable.^7^, ^9^, ^10^, ^11^ Our exploratory analysis suggests that most of these patients did require oxygen therapy. Although most did subsequently receive oxygen, this exposes a quality issue whereby prompt emergency treatment does not follow clinical detection. This delay could be due to many factors, including cost (eg, patients needing to pay before oxygen is provided), delayed prescription (eg, nurses having to wait for a doctor's prescription to administer oxygen), unavailability of oxygen equipment at admission, or patient or caregiver acceptance.^27^, ^31^ Future mixed-methods studies should explore how health-care workers make decisions about oxygen and how context-specific challenges to oxygen access can be addressed.

Our prevalence estimates for severe hypoxaemia were similar to those reported in medium-sized hospitals in Nigeria.^23^ Our data showed increased risk of mortality among neonates and children with moderate hypoxaemia (ie, SpO_2_ 90–93%) consistent with data from elsewhere.^28^, ^29^, ^32^ In settings where pulse oximetry is routinely done for acutely unwell children presenting to hospital, patients without SpO_2_ readings have a high risk of death.^32^ Health-care workers would be prudent in assuming that whenever they cannot obtain a pulse oximetry reading, patients have hypoxaemia and need urgent emergency care.

There are notable limitations to our study. First, it was an uncontrolled before–after intervention study, so we are unable to account for the effects of temporal factors. However, the large increases in pulse oximetry and oxygen service coverage, as well as their timing in relation to intervention activities, suggest that attribution of these effects to factors other than the intervention is unlikely. As health facilities in our study had improvements in pulse oximetry coverage and oxygen-service coverage that were equivalent to or higher than previous studies that have reported a mortality benefit for children, we consider applying these effect estimates to estimate effects reasonable.^7^, ^8^, ^9^, ^10^, ^11^ Second, we did not analyse COVID-19 pandemic variations in care seeking, oxygen supply, or illness severity as effectiveness outcomes were assessed among populations with the least risk of developing severe COVID-19.^33^ Third, we used routine patient records in the standard health-management information system reporting to the Ugandan Ministry of Health as our primary data source. Although pulse oximetry or oxygen therapy might have been provided without clinical documentation, we considered documentation to be an essential requirement of oxygen care. Furthermore, additional data-quality assurance measures included mentoring while working, supportive supervision, quality checks, and feedback. As expected a priori, this study was not powered to detect change in mortality.

The COVID-19 pandemic affected the study in several ways. First, health-facility attendance was low during the pre-intervention period due to restrictions involving suspended public transport and enforced curfews. Admission numbers increased as these restrictions were removed. Our pre-intervention and post-intervention populations were broadly similar and, although observed population differences (eg, age) were unlikely to substantially influence practice outcomes, they could have influenced mortality estimates. Second, the COVID-19 pandemic prompted additional attention to the oxygen crisis, which could have contributed to rapid increase in coverage of pulse oximetry and oxygen therapy.

This study shows that pulse oximetry and oxygen practices can be improved on a large scale through training; mentoring; provision of essential equipment; enhancing medical-oxygen supply chains; and clarifying policy, governance, and clinical-practice standards and guidelines. Large-scale improvements in health care and biomedical capacity to provide basic medical oxygen services are achievable, providing substantial improvements in patient access to pulse oximetry and oxygen in a relatively short timeframe. Our results should encourage other countries to develop national oxygen plans and to focus investment on interventions that have been shown to be effective, including the introduction of pulse oximetry into routine hospital care and clinical and biomedical mentoring and support.

### Contributors

### Equitable partnership declaration

### Data sharing

De-identified data are available upon request to the corresponding author and will be uploaded to a public data repository on completion of related publications, expected Dec 31, 2025. Data will be shared with other researchers for secondary analyses, systematic reviews, and meta-analyses with or without investigator support (depending on request) after approval of a proposal with a signed data access agreement. Additional related documents, including the study protocol and data-collection tools, will be made available with publication.

## Declaration of interests

HRG is an adviser to the Lifebox Foundation and Unitaid and receives a salary from the University of Melbourne. YK, JM, BK, SE, DK, AZ, LNK, and FL were employed by Clinton Health Access Initiative during implementation of the oxygen programme in Uganda. YK, BK, SE, AZ, DK, and LNK received a salary from Clinton Health Access Initiative Uganda. CO is employed by the Ugandan Ministry of Health. FEK and FS receive a salary from Makerere University. HA declares no competing interests.

## Supplementary Materials

Luganda translation of the abstract

Lusoga translation of the abstract

Supplementary appendix 3

Equitable Partnership Declaration
