# Supplementary material for: Improving effective coverage of medical-oxygen services for neonates and children in health facilities in Uganda: a before–after intervention study
Source: Lancet Glob Health. 2024 Aug 14;12(9):e1506–16. doi: 10.1016/S2214-109X(24)00268-7 (PMC11345447; doi:10.1016/S2214-109X(24)00268-7)
Supplement: Luganda translation of the abstract [file mmc1.pdf]

THE LANCET
Global Health

Supplementary appendix 1

This translation in Luganda was submitted by the authors and we reproduce it as supplied. It has not been peer reviewed. The Lancet's editorial processes have only been applied to the original in English, which should serve as reference for this manuscript.

Ekikyusiddwa mu Luganda kino kyawebwayo abawandiisi baakyo era netukivvuunula nga bwekyatuweebwa. Tekyekennenyezeddwa bantu balala (ba peers). Enkola y'okufulumya amawulire eya 'The Lancet' eteereddwa mu nkola mu kifulumziddwa mu Lungereza lwokka, era nga kino kimala okuwa obujulizi obwenkukunala eri ekiwandiiko kino.

Supplement to: Graham HR, Kitutu FE, Kamuntu Y, et al. Improving effective coverage of medical-oxygen services for neonates and children in health facilities in Uganda: a before–after intervention study. *Lancet Glob Health* 2024; **12:** e1506–16.

Omutwe:

Okulongoosa enkola ennuŋŋamu ey'okusaasaanya empeereza y'obujjanjabi bw'omukka gwa *okusigyeni* eri abaana abawere n'abaana mu bifo by'ebyobulamu mu Uganda: Ng'okunoonyereza tekunnaba-nga okunoonyereza kuwedde.

Abawandiisi:
Hamish R Graham*, Freddy Eric Kitutu*, Yewande Kamuntu, Blasio Kunihira, Santa Engol, Jasmine Miller, Absolom Zisanhi, Dorcas Kemigisha, Lorraine Nabbanja Kabunga, Charles Olaro, Harriet Ajilong, Freddie Ssengooba, Felix Lam

Ekifunze

Ensibuko: Omukka gwa *okusigyeni* mu by'obujjanjabi kikulu nnyo mu kujjanjaba abalwadde abayi. Okunoonyereza kuno kwatunuulira engeri okuyingira mu nsonga z'ebyobulamu gye kwakosa okuzuula abalwadde abalina omukka gwa *okusigyeni* omutono mu musaayi, okubawa obujjanjabi bwa *okusigyeni* bwe baba nga babwetaaga, n'okukendeeza ku kufa kw'abaana abawere n'abaana mu bifo by'ebyobulamu mu Busoga ne mu Mambuka ga Buganda mu Uganda.

Enkola: Twakuŋŋaanya ebyazuulibwa ku byatunuulirwa mu kunoonyereza omuli engeri z'abalwadde nga; emyaka, ekikula n'obubonero bw'endwadde zaabwe, wamu n'ebivaamu, mu balwadde abalina ebisaanyizo mu bifo by'ebyobulamu eby'okunoonyereza ng'okunoonyereza tekunnaba-ng'okunoonyereza kuwedde. Abalwadde abaalina ebisaanyizo kwaliko abaana abawere (abatasukka mwezi 1) n'abaana (abaweza omwezi 1 okutuuka ku myaka 14) abaaweebwa ebitanda mu bulwaliro obutono 24 n'amalwaliro 7 mu bitundu bya Busoga ne mu Mambuka ga Buganda mu Uganda okuva mu Gwomukaaga nga 1, 2020, okutuuka mu Gwomukaaga nga 30, 2022. Empeereza z'ebyobulamu mwe mwali okutendekebwa kw'abeebyobulamu nga baweebwa ebyuma ebikebera entunnunsi ebya *pulse oximeters* (ebikebera ekipimi ky'omukka gwa *okusigyeni* mu musaayi gw'omulwadde) wamu n'okulaba nga waliwo empeereza y'omukka gwa *okusigyeni* ogukozesebwa abasawo etatataaganyiziddwa. Ekipimo ekikulu ku byava mu kunoonyereza, kyali bitundu ku kikumi eby'abalwadde abaali bawandiikiddwa mu kusoma kw'ekyuma kya *pulse oximetry* ku lunaku olusooka nga baweereddwa ekitanda. Ebyava mu kunoonyereza by'ekkaanyizibwa nga bakozeza enkola ez'ebibalo.

Ebyazuulibwa: Ebyazuulibwa byakuŋŋaanyizibwa okuva mu baana abawere n'abaana 71,997 abalina ebisaanyizo mu bifo 31. Okwekkenneenya okukulu kwalimu abalwadde 10,001 nga tebannaba kuyingira mu nsonga (mu Gwomukaaga nga 1 okutuuka mu Gwekkumi nga 30, 2020) n'abalwadde 51,329 oluvannyuma lw'okuyingira mu nsonga (mu Gwokusatu nga 1, 2021, okutuuka mu Gwomukaaga nga 30, 2022). Okukozesa kw'ekyuma kya *pulse oximetry* nga abalwadde bayingizibwa mu ddwaliro kweyongera okuva ku 23.7% (2,365 ku 10,001) nga tebannaba kuyingira mu nsonga okutuuka ku 87.7% (45,029 ku 51,328) oluvannyuma lw'okuyingira mu nsonga. Okwekkenneenya kwalaga nti emikisa mingi nnyo egy'okukozesa *pulse oximetry* oluvannyuma lw'okuyingira mu nsonga (odds ratio 40.10, 95% CI 37.38–42.93; $p<0.0001$).

Okuvvuunula: Ennongoosereza ennene mu mpeereza y'omukka gwa *okusigyeni* mu by'obujjanjabi mu malwaliro kisoboka era kiyinza okuvaako ebiva mu balwadde ebirungi. Gavumenti zirina okukola enteekateeka z'eggwanga ez'omukka gwa *okusigyeni* n'okussa ssente mu bikolwa ebikakasibwa nga okukozesa bulijjo *pulse oximetry* n'okutendeka abakozi b'ebyobulamu, enkola za *okusigyni* ez'obujjanjabi ezinywezeddwa nga zigatta wamu.
