# Supplementary material for: Improving effective coverage of medical-oxygen services for neonates and children in health facilities in Uganda: a before–after intervention study
Source: Lancet Glob Health. 2024 Aug 14;12(9):e1506–16. doi: 10.1016/S2214-109X(24)00268-7 (PMC11345447; doi:10.1016/S2214-109X(24)00268-7)
Supplement: Lusoga translation of the abstract [file mmc2.pdf]

THE LANCET
Global Health

## Supplementary appendix 2

This translation in Lusoga was submitted by the authors and we reproduce it as supplied. It has not been peer reviewed. The Lancet's editorial processes have only been applied to the original in English, which should serve as reference for this manuscript.

Okucuusibwa kuno mu Lusoga kwaleetebwa abaghandiisi, ife tukucuusizaamu nga bwebaakireeta. Tikisoose kwekeneenezebwamu. Okwekeneeniamu ni ba Lancet kwakolebwa ku bughandiike bule bwene obwasooka mu Lungereza era n'okunaakola nga ekyesigamwaku ni mu kighandiiko kino eky'Olusoga

Supplement to: Graham HR, Kitutu FE, Kamuntu Y, et al. Improving effective coverage of medical-oxygen services for neonates and children in health facilities in Uganda: a before–after intervention study. *Lancet Glob Health* 2024; **12:** e1506–16.

**Omutwe:**
**Okulongosaamu enkola enungamu mu mu bwidhandhabi ni  oxyen eri neonates n’abaana abali mu bifo ebidhandhabibwamu mu Uganda : a nga ghakaali kubaagho – n’oluvainhuma lwokubaagho omusomo ogulungamya enkola eno .**

**Abaghandiisi :**
Hamish R Graham*, Freddy Eric Kitutu*, Yewande Kamuntu, Blasio Kunihira, Santa Engol, Jasmine Miller, Absolom Zisanhi, Dorcas Kemigisha, Lorraine Nabbanja Kabunga, Charles Olaro, Harriet Ajilong, Freddie Ssengooba, Felix Lam

**Mu bufunze**

**Ebikigemaku** : okwidhandhabwa n’omuka gw’obulamu kya mughendo inho eri abalwaire abayi.  Omusomo guno gwalinga ku ngeri yokuyambamu abo abalwaire abagaanibwa nga bali ni puleesa owaghansi , okubagha omuuka ogugemerera okugheera mwirwaliro bwebaba bagwetaaga , n’okukendeeza ku nfa y’abaana abakazaalibwa n’abaana ababa mu bifo ebidhandhabirwamu mu  Busoga ni mu mambuka g’ekiketezo kya Buganda mu Uganda.

**Engeri  yekyakolebwamu : Twasoloza ebintu ebyendhawulo binji omwali embeera dh’abalwaire katukobe emyaka gy’obukulu, ekikula ky’omuntu n’obumanhiso bw’obulwaire obumuliku, n’ebivaamu ku balwaire abaalondebwamu okukolebwaku mu bifo ebidhandhabirwamu ebyatongozebwa nga okulongosaamu enkola eno kukaali kubaagho n’oluvainhuma lwokugikozesa.**  Abalwaire abaalondebwamu n’omwali abaana abakazaalibwa  (abali wansi w’omwezi 1 } n’abaana  (aboomwezi 1 okutuusa ku baana b’emyaka 14 ) bano n’abagheebwa ebitanda mu bulwaliro obutono 24 ni mu malwaliro amanene 7 mu Busoga ni mumambuka g’ekiketezo kya Buganda mu Uganda okuva nga enaku 1 mu mwezi gwa Namwendwa 2020,okutuusa ku nnaku 30 mu mwezi gwa Namweandwa 2022. Engeri yokulongosaamu endhidhandhaba enungamu eno n’omwali okwegesa abasawo, okufuna obuuma obupima entununsi y’omutima,  (n’ebyuma ebindi ebipima obunji bw’omusaayi mu mulwaire  ), n’obunji bw’abalwaire abaali bali n’ebighandiiko ebiraga bwebaababapimanga  ku lunaku lwebabagheerwaku ekitanda  n’ebyavaamu twakozesa bibalo ebiraga buli mutendero.

**Ebyavaamu** : Ebyavaamu byateebwa aghalala okuva ku baana abakazaalibwa 71,997 n’abaana abaali mu bifo ebidhandhabirwamu 31.  Ekikulu ekyanhunkuzibwa n’omwali abalwaire 10,001 abaaligho nga endhidhandhaba enongoseemu ekaali kukozesebwa (omwezi gwa Namwendwa nga 1 okutuusa nga Namutyabuko 30, 2020) n’abalwaire abandi 51,329  oluvainhuma lwokwidhandhabwa  (mu mwezi gwa Mulyaiva nga enaku 1, 2021, okutuusa mu mwezi gwa Namwendwa nga enaku 30, 2022). Okukozesa akooma akapima entununsi mu kubagha ekitanda kweyongeramu okuva ku 23.7% (2,365 ku 10,001) nga bakaali kukolebwaku okutuusa ku 87.7% (45,029 ku 51,328) oluvainhuma lwokukolebwaku Ekyavaamu kyalaga kiti waaligho okweyongeramu obunji bw’abalwaire abaakolebwaku nga tukozesa akooma kano akapima entununsi y’omutima (endhawulo eyaligho  40.10, 95% CI 37.38–42.93; $p<0.0001$).

**Kyekitegeeza** : Okwidhandhabwa n’ekyuma ekighanirira okwisa omuuka kweyongeramu inho mu malwaliro webwasobola okubanga ekisobozesa embeera dh’abalwaire okulongooka mu bwangu.  Gavumenti esaine eteegho enkola yokukozesa omuuka ogughanirira okwisa omuuka nkani okugheera  mu Uganda yoonayoona nga bataamu amaani mu nnongekereza eno ekakasiibwa nga bakozesa akooma kano akapima entununsi buli kiseera aghalala n’okusomesa abasawo abakola ku by’obulamu,n’okwongeramu obunji  bw’ebyuma bino ebipima.
