# Supplementary material for: Improving effective coverage of medical-oxygen services for neonates and children in health facilities in Uganda: a before–after intervention study
Source: Lancet Glob Health. 2024 Aug 14;12(9):e1506–16. doi: 10.1016/S2214-109X(24)00268-7 (PMC11345447; doi:10.1016/S2214-109X(24)00268-7)

THE LANCET
Global Health

Supplementary appendix 3

This appendix formed part of the original submission and has been peer reviewed. We post it as supplied by the authors.

Supplement to: Graham HR, Kitutu FE, Kamuntu Y, et al. Improving effective coverage of medical-oxygen services for neonates and children in health facilities in Uganda: a before–after intervention study. *Lancet Glob Health* 2024; **12:** e1506–16.

Supplemental material

Improving effective coverage of medical oxygen services for neonates and children in health facilities in Uganda: a before-after interventional study.

Contents

Supplemental material 1

Table S1 Overview of the levels of health facilities in Uganda, key governance agencies, and typical health services provided. 2

Table S2 Description of the multi-level multi-component health system intervention to improve medical oxygen service coverage in Uganda. 3

Figure S1 Study participant flow chart 6

Figure S2 Admission numbers over time, by facility type (June 2020 to June 2022) 7

Table S3 Characteristics of the study health facilities during the study period 8

Table S4 Participant characteristics, extended table by age group 9

Table S5 Effect of oxygen systems strengthening activities on pulse oximetry and oxygen practices and in-hospital mortality, by age group 11

Table S6 Extrapolated additional patients served with pulse oximetry and oxygen, and deaths averted, during the post-intervention period 13

Table S7 Exploratory analysis of subpopulations and their likelihood of receiving oxygen and odds of death (restricted to post-intervention period Jul 2021 to Jun 2022) 14

Table S8 Exploratory analysis of those with severe hypoxaemia (SpO2<90%) who do not receive oxygen on admission (restricted to post-intervention period Jul 2021 to Jun 2022) 14

Figure S3 Pulse oximetry coverage (proportion of admissions with documented SpO_2) over time, by age group and facility type 15

Figure S4 Oxygen coverage to hypoxaemic patients (SpO_2<90% or clinical signs of hypoxaemia if no pulse oximetry), by age group and facility type 16

Figure S5 Oxygen usage to all patients, by age group and facility type 18

Table S1 Overview of the levels of health facilities in Uganda, key governance agencies, and typical health services provided.

<table>
<tr><th>Governance</th><th colspan="2">Health unit and scope</th><th>Implication for medical oxygen system</th></tr>
<tr><td>Parliament</td><td colspan="2">Ministry of Health, Departments and Agencies</td><td>Inter-departmental collaboration among clinical services, pharmaceuticals and natural medicines, health infrastructure and emergency medical services at MoH. Coordinates strategic planning, resource mobilisation and policy and standards development and dissemination, including public-private partnerships for medical oxygen systems.</td></tr>
<tr><td rowspan="2">National and Regional Level Hospital Boards</td><td colspan="2">National referral hospitals</td><td>Provide medical oxygen services at the health facility and operates health facility-based Pressure Swing Adsorption (PSA) to manufacture and distribute of medical oxygen for its own use and to neighbouring health facilities.</td></tr>
<tr><td colspan="2">Regional referral hospitals</td><td>Provides medical oxygen services at the health facility and operates health facility-based Pressure Swing Adsorption (PSA) to manufacture and distribute of medical oxygen for its own use and to neighbouring health facilities. Provides technical guidance and supportive supervision to lower-level health facilities within their catchment area.</td></tr>
<tr><td rowspan="6">Ministry of Local Government, District Councils, Chief Administrative Officer, District Health Teams</td><td colspan="2">District local government</td><td>Oversee health service provision in lower-level health facilities within the district including coordinating medical oxygen supply and distribution, supportive supervision, and resource mobilisation.</td></tr>
<tr><td colspan="2">General hospitals</td><td>Provide medical oxygen services at the health facility, technical guidance, and supportive supervision to lower-level health facilities within their catchment area.</td></tr>
<tr><td colspan="2">Level IV health centre</td><td>Provide medical oxygen services at the health facility, technical guidance and supportive supervision to lower-level health facilities within their catchment area</td></tr>
<tr><td colspan="2">Level III health centre</td><td>Provide medical oxygen services at the health facility</td></tr>
<tr><td rowspan="2">Sub-Districts</td><td>Level II health centre</td><td>Provide outpatient services only and may offer pulse oximetry screening.</td></tr>
<tr><td>Village health teams (VHT)</td><td>Health promotion and preventive care including addressing community perceptions about care-seeking and use of medical oxygen.</td></tr>
</table>

Table S2 Description of the multi-level multi-component health system intervention to improve medical oxygen service coverage in Uganda.

Intervention component	Detailed activities
**National level**	
National scale-up of medical oxygen implementation plan	The Ministry of Health through a multi-stakeholder process developed and implemented the National Scale-up of Medical Oxygen Implementation Plan 2018/19-2022/23. It prioritized creating a strategic framework for medical oxygen supply chain and utilization, emphasis on maintenance, repair or replacement of medical oxygen equipment, capacity building for health workers and biomedical personnel on pulse oximetry, oxygen therapy and routine maintenance for medical oxygen systems, and advocacy for resource mobilization.
National Committee on Medical Equipment guidelines	The national medical equipment guidelines were reviewed to include specifications for medical oxygen equipment and procurement guidelines and disseminated to all relevant stakeholders.
National Oxygen therapy guidelines and essential medicines and health supplies list	Clinical guidelines for hypoxemia detection and oxygen therapy were developed and disseminated. Additionally, the medical oxygen and related consumables were added to the national essential medicines and health supplies list. Based on these policy documents, CHAI supported to MOH to hold annual forecasting and budgeting workshops to review and revise oxygen-related budgets for health facilities.
**District level**	
Orientation and coordination with district level leadership	The district health teams responsible for the lower-level health facilities (General hospitals and level IV health centers) in the 16 study districts were engaged and their roles in the multilevel multicomponent intervention was elaborated. They participated in support supervision, coordination of the hub and spoke distribution model of medical oxygen and resource mobilisation. The also participated nomination and selection of healthcare workers (HCWs) from health facilities as Oxygen Mentors/Champions based on previous mentoring and teaching experience, motivation and ability to cascade skills to colleagues, and perceived influence to effect change.
**Health facility level**	
Provision of catalytic medical oxygen equipment, their maintenance, refurbishment, and power supply.	Health facilities were categorised according to existing readiness to provide medical oxygen services based in a baseline assessment of health worker capacity, available functional oxygen related equipment. CHAI provided financial and logistic support for catalytic procurement of pulse oximeters, oxygen concentrators, oxygen cylinders and related accessories. This enabled health facilities with some pulse oximeters to get more and those without any to get their first pulse oximeters. All study health facilities had been supplied with pulse oximeters by January 2021. CHAI provided complementary support to increase sustainable availability of medical oxygen at study health facilities by improving on the medical oxygen infrastructure in place. CHAI refurbished Pressure Swing Adsorption (PSA) oxygen plants at the two regional referral hospitals (RRH) that serve both these hospitals and smaller health facilities within the catchment area. Spare parts were procured and made available to the plant operators and biomedical engineers. When these PSA oxygen plants were not operational, health facilities were supported to get their oxygen supply from nearby RRH

	(Hoima and Fort Portal for Buganda North region) or private firms such as and Tembo and Pramukh industries for the Busoga region.
Healthcare worker capacity to provide medical oxygen services	To improve HCW practices, CHAI program staff developed a hypoxaemia education and mentoring program and supported based on findings of the needs assessment. CHAI worked with a multi-disciplinary team of health professionals including paediatricians, intensivists, obstetrician, clinical pharmacist, nurse and midwives from MOH and professional associations to develop the curriculum and ensure consistency with national guidelines. Oxygen Mentors/Champions from participating health facilities were selected based on previous mentoring and teaching experience, motivation and ability to cascade skills to colleagues, and perceived influence to effect change.<br>First, the health workers participated in a 5-day, centrally based Oxygen mentoring workshop led by CHAI program staff and clinical experts (National Mentors) using didactic and participatory teaching methods, including group discussion, scenarios, assessment tasks, participant manuals, and daily practice at the nearby RRH. Second, on-site training and mentorship sessions were conducted by health facility oxygen champions, supported by mentors and supervisors operating at regional and national levels, respectively. HCWs who excelled at the mentoring workshop were given additional responsibility to mentor peers in other health facilities within the region while others were designated as Oxygen Champions at their home facility. Regional Mentors joined the National Mentors to conduct onsite mentorship to participating facilities, with each Mentor allocated 2 facilities and expected to visit at 2-3 months intervals. Oxygen Champions in each facility acted as the key focal person for mobilising HCWs for onsite training and mentorship, participating in data audit and feedback, and directly supervised and encouraged HCWs to adopt pulse oximetry and oxygen-related practices (including documentation). During mentoring visits, the Mentor and Champion would engage individual HCWs in practice-based observation and discussion using a competency booklet as a guide, with the Champion continuing similar activities in between visits. In addition to imparting knowledge and skills, these sessions incorporated data audit and feedback, supervision and initiation of recommenced pulse oximetry and oxygen therapy, patient and oxygen record practices. All Mentors and Champions received financial allowances for travel and attendance and of meetings but were not paid for their services. Third, representative health workers from study health facilities participated in quarterly "best practice" workshops intended to promote knowledge exchange, cross health facility learning, share challenges, and discuss possible solutions. Priority health facilities where poor performance issues had persisted were identified for remedial action that would be led by the MoH national quality improvement team.
Biomedical engineer / technician capacity to provide medical oxygen services	To build the capacity of biomedical technicians (BMET), CHAI program staff provided technician training, implemented a new equipment inventory system, introduced an oxygen distribution system, and procured additional oxygen-related equipment and tools. Capacity building for biomedical engineers and technicians was done through a workshop based on an adapted Biomedical Engineering and Technician oxygen training curriculum. It was delivered by master trainers for one day in each of the regions. The

	engineers and technician were taught and shown how to maintain and repair of oxygen-related equipment – oxygen concentrators, pulse oximeters and pressure swing adsorption oxygen plants. CHAI went further to supplement the existing staff by collaborating with the health facilities to employ plant operators and pay their salaries. The workshop was complemented by external supervision in on-site visits, consultations and remote support via platforms such as *WhatsApp*
**Vertical components**	
Enhancing the medical oxygen supply chain	CHAI worked with the National Medical Store (NMS), - the public entity mandated to procure, warehouse, and distribute essential medicines, health supplies and equipment to public health facilities with public funds – to set up a reliable oxygen supply system. This included supporting NMS to identify oxygen producers, acquiring additional cylinders and setting up a cylinder exchange system with health facilities on delivery of medical oxygen. CHAI program staff and experts from the Centre for Public Health and Development (CPHD) supported the MOH to design and pilot a regional hub-and-spoke model for distributing oxygen cylinders from the RRH PSA plants to smaller facilities. In this model, a single private logistics provider managed the ordering and supply of oxygen cylinders from the RRH (supplemented from the National Medical Store, NMS, and private oxygen providers as needed) to smaller facilities. This was intended to be implemented nationally but was terminated prematurely due to COVID-19 surge and only operated from during March to June 2021 in Jinja and Mubende regions, subsequently replaced with the National medical oxygen distribution system. This included processes for refilling and distributing cylinders and introduction of cylinder exchange for the first time, as well as procurement of additional cylinders to strengthen the supply chain back bone and improve its capacity. CHAI/MOH encouraged free cylinder filling at both the public RRH PSA plants and at the private industries and assisted with oxygen cylinder transportation. Eventually the National Medical Stores (NMS), the country's health commodity procurement and distribution agency, was looped into some the core components of the piloted medical oxygen supply chain with intention to scale up and enhance sustainability.
Building the capacity to provide biomedical support to oxygen systems	CHAI also worked with the MoH department of infrastructure and medical equipment to advocate for recruitment of more biomedical personnel at MoH and at regional referral and district hospitals, revitalizing regional biomedical workshops by identifying and addressing bottlenecks of medical equipment servicing and maintenance such as lack of spare parts and implementation of the *New Order for Managing Anything Data (NOMAD)* equipment inventory database software to track available and functional medical oxygen related equipment. The NOMAD software was rolled out nationally to all regional BMET workshops, including Jinja and Mubende through on-site training and demonstration. This made it easier for engineers to manage all their equipment, devices and spare parts – applicable well beyond oxygen service provision. CHAI also provided tools and spare parts to the two regional workshops in Jinja and Mubende.

Figure S1 Study participant flow chart

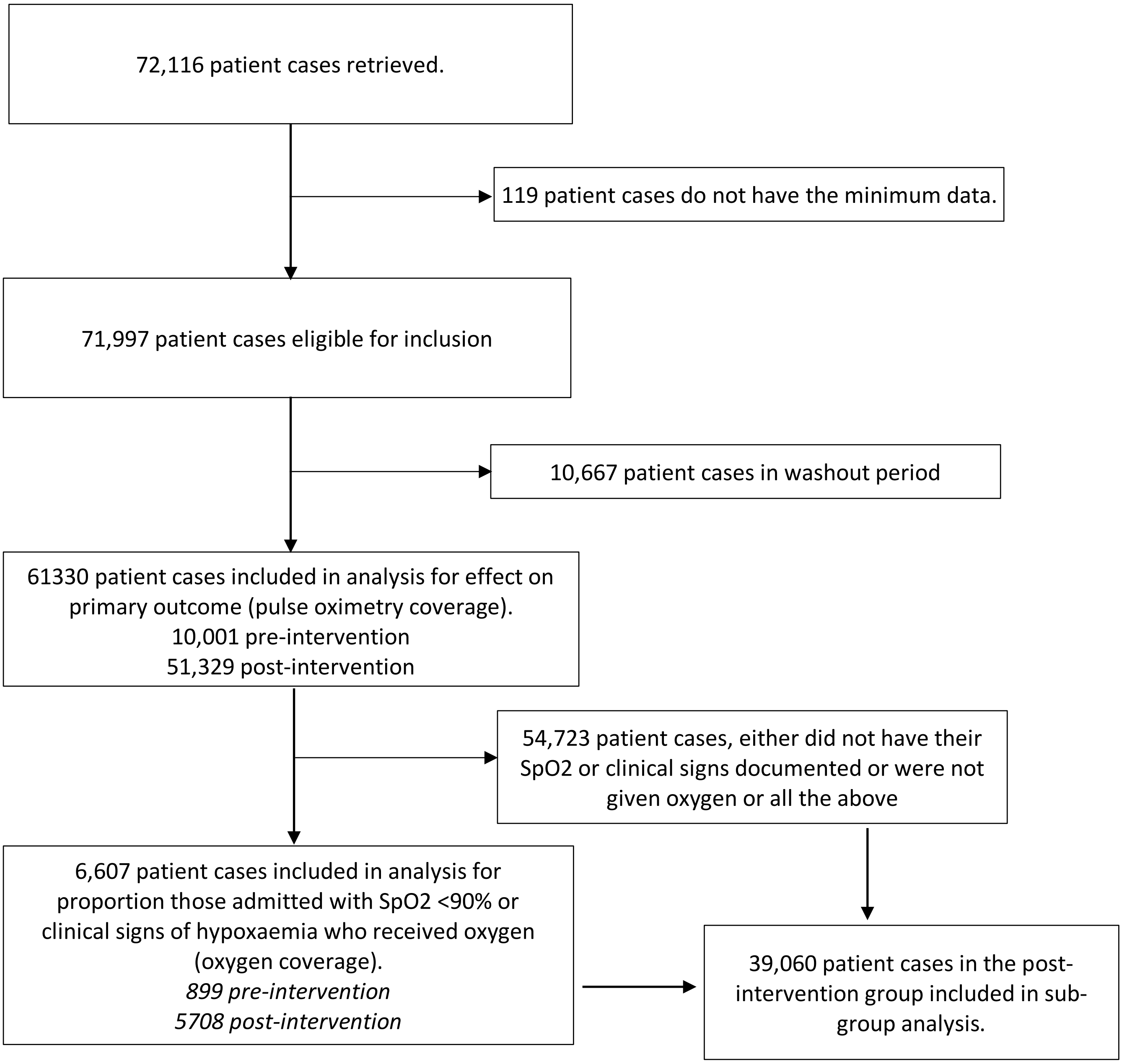

Figure S2 Admission numbers over time, by facility type (June 2020 to June 2022)

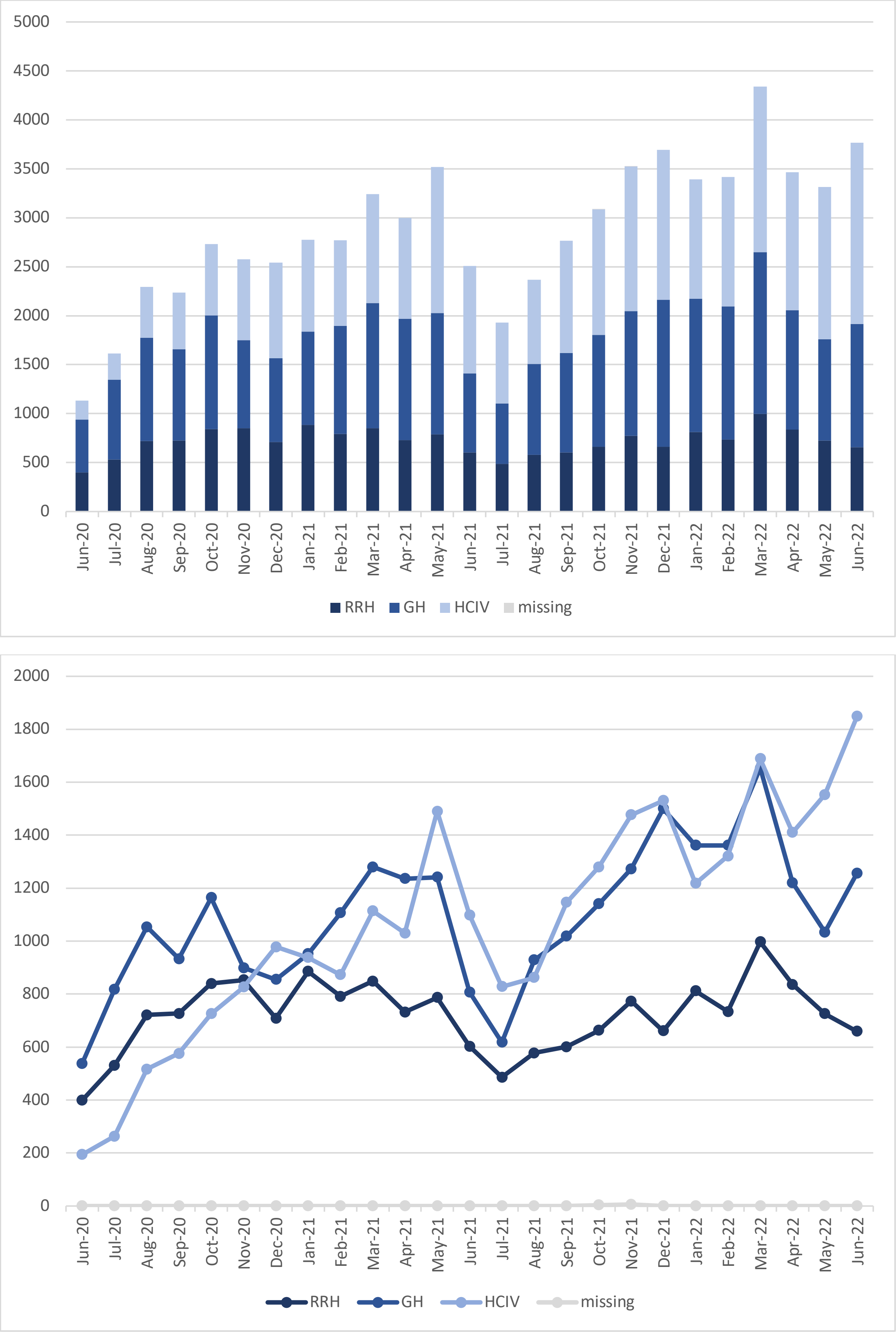

Table S3 Characteristics of the study health facilities during the study period

Variable	Level of care		
	Health centre IV	General hospital	Regional referral hospital
Number of health facilities (N=31)	24	5	2
Number of patients (N=71997)	26788 (37%)	27255 (38%)	17954 (25%)
Sex distribution			
Female	11910 (48.2%)	12315 (46.5%)	7650 (43.7%)
Male	12815 (51.8%)	14168 (53.5%)	9864 (56.3%)
Age distribution			
Neonates (aged < 1 month)	3515 (13.6%)	8804 (32.8%)	5497 (31.8%)
Infants (aged 1 – 11 months)	3375 (13.0%)	3490 (13.0%)	3026 (17.5%)
Young child (aged 12 – 59 months)	12401 (47.9%)	9455 (35.2%)	5641 (32.7%)
Older child (aged 5 – 9 years)	4717 (18.2%)	3970 (14.8%)	2259 (13.1%)
Young adolescent (aged 10 – 14 years)	1879 (7.3%)	1133 (4.2%)	847 (4.9%)

Table S4 Participant characteristics, extended table by age group

NEONATES (aged <1 month)	Pre-intervention (Jun-Oct 2020) N=3177	Post-intervention (Mar 2021-Jun 2022) N=12,393	P-value
Median gestation, weeks (25th to 75th centile)	38 (35-39)	38 (34-38)	<0.001
Sex, female (%)	1255 (44.0%)	5176 (44.9%)	0.398
Region, Mubende (%)	1219 (38.4%)	4625 (37.3%)	0.275
Health centre IV	409 (12.9%)	2706 (21.8%)	<0.001
General Hospital	1692 (53.3%)	6180 (49.9%)	
Regional Referral Hospital	1076 (33.9%)	3507 (28.3%)	
**Presenting signs and symptoms**			
Fever / chills / shivering	543 (17.1%)	2727 (22.0%)	<0.001
Cough	38 (1.20%)	239 (1.9%)	0.005
Fast / difficult breathing	627 (19.7%)	3154 (25.5%)	<0.001
Chest indrawing	71 (2.23%)	566 (4.6%)	<0.001
Severe respiratory distress (cyanosis, grunting, nasal flaring, stridor)	424 (13.4%)	2528 (20.4%)	<0.001
Other respiratory (wheeze, noisy, slow)	15 (0.47%)	168 (1.4%)	<0.001
Diarrhoea	17 (0.54%)	29 (0.2%)	0.005
Altered conscious state (drowsy, unconscious)	805 (25.3%)	3253 (26.3%)	0.297
Seizures	113 (3.56%)	540 (4.4%)	0.045
Pain (abdo, other, infant grimace)	44 (1.38%)	260 (2.1%)	0.01
Poor feeding / drinking (incl vomiting)	575 (18.1%)	3005 (24.3%)	<0.001
Mean heart rate (SD)	129 (24)	132 (23)	<0.001
Mean respiratory rate (SD)	50.7 (13)	47.7 (11)	<0.001
Signs of hypoxaemia (respiratory distress, chest indrawing, cyanosis, grunting, nasal flaring, drowsy, unconscious)	454 (14.3%)	2722 (22.0%)	<0.001
WHO Emergency signs (altered consciousness, severe respiratory distress, uanable to feed/drink, chestindrawing in older child)	1453 (45.7%)	6622 (53.4%)	<0.001
Hypoxaemia (SpO2<90%)	391/1227 (31.9%)	3248/11374 (28.6%)	0.015
Moderate hypoxaemia (SpO2 90-93%)	269/1227 (21.9%)	1736/11374 (15.3%)	<0.001
**Admission diagnoses - neonatal**			
Neonatal encephalopathy	1073 (33.8%)	4195 (33.9%)	0.936
Neonatal sepsis	879 (27.7%)	4135 (33.4%)	<0.001
Small / preterm	913 (28.7%)	4033 (32.5%)	<0.001
Jaundice	107 (3.4%)	575 (4.6%)	0.002

CHILDREN (aged 1 month to 14 years)	Pre-intervention (Jun-Oct 2020)	Post-intervention (Mar 2021-Jun 2022)	P-value

	N=6824	N=38,936	
Median age, months (25th to 75th centile)	30 (14-60)	30 (14-60)	0.414
Sex, female (%)	3110 (47.9%)	17655 (46.6%)	0.057
Region, Mubende (%)	2055 (30.1%)	10892 (28.0%)	<0.001
Health centre IV	1867 (27.4%)	18191 (46.7%)	<0.001
General Hospital	2815 (41.3%)	12755 (32.8%)	
Regional Referral Hospital	2142 (31.4%)	7990 (20.5%)	
**Presenting signs and symptoms**			
Fever / chills / shivering	5311 (77.8%)	31282 (80.3%)	<0.001
Cough	2331 (34.2%)	19753 (50.7%)	<0.001
Fast / difficult breathing	825 (12.1%)	7125 (18.3%)	<0.001
Chest indrawing	90 (1.32%)	247 (0.6%)	<0.001
Severe respiratory distress (cyanosis, grunting, nasal flaring, stridor)	96 (1.41%)	666 (1.7%)	0.071
Wheeze	*26 (0.38%)*	*198 (0.5%)*	*0.164*
Other respiratory (wheeze, noisy, slow)	308 (4.51%)	1780 (4.6%)	0.832
Diarrhoea	1211 (17.8%)	8600 (22.1%)	<0.001
Altered conscious state (drowsy, unconscious)	128 (1.88%)	514 (1.3%)	<0.001
Seizures	615 (9.01%)	3277 (8.4%)	0.104
Pain (abdominal, other, infant grimace)	1550 (22.7%)	11665 (30.0%)	<0.001
Poor feeding / drinking (including vomiting)	2668 (39.1%)	14141 (36.3%)	<0.001
Dehydration	48 (0.70%)	381 (0.74%)	0.004
Mean heart rate (SD)	119 (27)	118 (26)	0.5169
Mean respiratory rate (SD)	32.4 (13)	32.7 (12)	0.5838
Signs of hypoxaemia (respiratory distress, chest indrawing, cyanosis, grunting, nasal flaring, drowsy, unconscious)	276 (4.04%)	1208 (3.1%)	<0.001
WHO Emergency signs (altered consciousness, severe respiratory distress, unable to feed/drink, chest in drawing in older child)	2807 (41.1%)	14919 (38.3%)	<0.001
Hypoxaemia (SpO2<90%)	127/1138 (11.2%)	2202/33655 (6.5%)	<0.001
Moderate hypoxaemia (SpO2 90-93%)	100/1138 (8.79%)	2750/33655 (8.2%)	0.456
**Admission diagnoses - post-neonatal**			
Malaria	4264 (62.5%)	22764 (58.5%)	<0.001
Pneumonia	687 (10.1%)	5865 (15.1%)	<0.001
Diarrhoeal disease	1211 (17.8%)	8600 (22.1%)	<0.001
Sepsis	1016 (14.9%)	6148 (15.8%)	0.059
WHO pneumonia	361 (5.3%)	3471 (8.9%)	<0.001
WHO severe pneumonia	186 (2.7%)	1879 (4.8%)	<0.001

Table S5 Effect of oxygen systems strengthening activities on pulse oximetry and oxygen practices and in-hospital mortality, by age group

**OVERALL (neonates and children)**	**n/N (%)***	**Mixed-model aOR (95% CI)**		**ICC (95% CI)**
		**Primary analysis**	**Extended model**	
**Primary outcome: Pulse oximetry coverage (proportion with documented SpO_2 on day 1 of admission)**				
Pre-intervention	2365/10001 (23.7%)			
Post-intervention	45029/51329 (87.7%)	40.1 (37.4-42.9)	39.2 (36.5-42.0)	0.26 (0.17-0.37)
**Oxygen coverage (oxygen to those with SpO_2<90%, or clinical signs of hypoxaemia if pulse oximetry not done)**				
Pre-intervention	358/899 (39.8%)			
Post-intervention	4074/5708 (71.4%)	3.81 (3.26-4.46)	3.90 (3.32-4.59)	0.09 (0.05-0.16)
**Oxygen usage (oxygen to anyone)**				
Pre-intervention	788/10001 (7.9%)			
Post-intervention	5789/51329 (11.3%)	1.95 (1.79-2.13)	1.73 (0.57-1.91)	0.12 (0.07-0.18)
**Oxygen coverage (oxygen to those with documented SpO_2<90%)**				
Pre-intervention	277/518 (53.5%)			
Post-intervention	3990/5450 (73.2%)	2.15 (1.77-2.63)	2.06 (1.68-2.53)	0.12 (0.07-0.20)
**Oxygen coverage (oxygen to those with clinical signs of hypoxaemia)**				
Pre-intervention	259/730 (35.5%)			
Post-intervention	2354/3930 (59.9%)	2.43 (2.01-2.94)	2.42 (1.98-2.94)	0.20 (0.12-0.32)
**Appropriate use of oxygen (proportion of those receiving oxygen who had SpO_2<90% or clinical signs of hypoxaemia if pulse oximetry not done)**				
Pre-intervention	358/788 (45.4%)			
Post-intervention	4074/5789 (70.4%)	3.18 (2.69-3.75)	2.91 (2.45-3.44)	0.20 (0.12-0.32)
**All-cause mortality (in-hospital death)**				
Pre-intervention	344/9043 (3.80%)			
Post-intervention	1560/49361 (3.16%)	1.06 (0.93-1.20)	0.93 (0.82-1.07)	0.23 (0.13-0.37)

**NEONATES (aged <1 month)**	**n/N (%)**	**Mixed-model aOR (95% CI)**		**ICC (95% CI)**
		**Primary analysis**	**Extended model**	
**Primary outcome: Pulse oximetry coverage (proportion with documented SpO_2 on day 1 of admission)**				
Pre-intervention	1227/3177 (38.6%)			
Post-intervention	11374/12393 (91.8%)	20.18 (18.07-22.5)		0.14 (0.08-0.23)
**Oxygen coverage (oxygen to those with SpO_2<90%, or clinical signs of hypoxaemia if pulse oximetry not done)**				
Pre-intervention	275/575 (47.8%)			
Post-intervention	2553/3411 (74.9%)	3.02 (2.48-3.69)		0.14 (0.07-0.23)
**Oxygen usage (oxygen to anyone)**				
Pre-intervention	587/3177 (18.5%)			
Post-intervention	3556/12393 (28.7%)	1.84 (1.65-2.04)		0.11 (0.07-0.18)
**Oxygen coverage (oxygen to those with documented SpO_2<90%)**				
Pre-intervention	209/391 (53.5%)			
Post-intervention	2472/3248 (76.1%)	2.29 (1.82-2.90)		0.23 (0.13-0.37)
**Oxygen coverage (oxygen to those with clinical signs of hypoxaemia)**				

Pre-intervention	222/454 (48.9%)			
Post-intervention	1878/2722 (69.0%)	2.28 (1.82-2.85)		0.14 (0.07-0.25)
**Appropriate use of oxygen (proportion of those receiving oxygen who had SpO_2<90% or clinical signs of hypoxaemia if pulse oximetry not done)**				
Pre-intervention	275/587 (46.9%)			
Post-intervention	2553/3556 (71.8%)	3.14 (2.57-3.84)		0.26 (0.15-0.40)
**All-cause mortality (in-hospital death)**				
Pre-intervention	224/2870 (7.80%)			
Post-intervention	976/11878 (8.22%)	1.14 (0.97-1.34)		0.09 (0.04-0.19)
**CHILD (aged 1 month to 14 years)**	**n/N (%)**	**Mixed-model aOR (95% CI)**		**ICC (95% CI)**
		**Primary analysis**	**Extended model**	
**Primary outcome: Pulse oximetry coverage (proportion with documented SpO_2 on day 1 of admission)**				
Pre-intervention	1138/6824 (16.7%)			
Post-intervention	33655/38936 (86.4%)	84.8 (76.5-93.9)		0.41 (0.29-0.56)
**Oxygen coverage (oxygen to those with SpO_2<90%, or clinical signs of hypoxaemia if pulse oximetry not done)**				
Pre-intervention	83/25.6 (25.6%)			
Post-intervention	1521/2297 (66.2%)	6.40 (4.80-8.52)		0.12 (0.06-0.20)
**Oxygen usage (oxygen to anyone)**				
Pre-intervention	201/6824 (3.0%)			
Post-intervention	2233/38936 (5.7%)	2.31 (2.00-2.69)		0.21 (0.13-0.31)
**Oxygen coverage (oxygen to those with documented SpO_2<90%)**				
Pre-intervention	68/127 (53.5%)			
Post-intervention	1518/2202 (68.9%)	1.69 (1.12-2.55)		0.15 (0.08-0.25)
**Oxygen coverage (oxygen to those with clinical signs of hypoxaemia)**				
Pre-intervention	37/276 (13.4%)			
Post-intervention	476/1208 (39.4%)	3.15 (2.06-4.80)		0.40 (0.24-0.58)
**Appropriate use of oxygen (proportion of those receiving oxygen who had SpO_2<90% or clinical signs of hypoxaemia if pulse oximetry not done)**				
Pre-intervention	83/201 (41.3%)			
Post-intervention	1521/2233 (68.1%)	3.14 (2.57-3.84)		0.26 (0.15-0.40)
**All-cause mortality (in-hospital death**				
Pre-intervention	120/6173 (1.94%)			
Post-intervention	584/37483 (1.56%)	0.94 (0.76-1.16)		0.36 (0.21-0.55)
**Child pneumonia mortality (in-hospital death)**				
Pre-intervention	27/513 (5.26%)			
Post-intervention	188/5205 (3.61%)	0.70 (0.46-1.08)	0.80 (0.51-1.23)	0.23 (0.10-0.45)

Primary analysis model adjusted for age, sex, and facility. Extended analysis model adjusted for age, sex, facility PLUS region, health facility level, cough, fast/difficult breathing, chest indrawing, convulsions, altered conscious state, fever, ability to feed/drink, severe respiratory distress

CI - confidence interval; ICC - intracluster correlation coefficient; OR - odds ratio.

Table S6 Extrapolated additional patients served with pulse oximetry and oxygen, and deaths averted, during the post-intervention period

	Effect size	Denominator	Observed	Counterfactual	Difference
**Primary outcome: Pulse oximetry coverage (proportion with documented SpO_2 on day 1 of admission)**					
Overall	**40.1**	51329	45029	1123	-43906
Neonate	20.18	12393	11374	564	-10810
Child	84.8	38936	33655	397	-33258
**Oxygen coverage (oxygen to those with SpO_2<90%, or clinical signs of hypoxaemia if pulse oximetry not done)**					
Overall	**3.81**	5708	4074	1069	-3005
Neonate	3.02	3411	2553	845	-1708
Child	6.4	2297	1521	238	-1283
**Oxygen usage (oxygen to anyone)**					
Overall	**1.95**	51329	5789	2969	-2820
Neonate	1.84	12393	3556	1933	-1623
Child	2.31	38936	2233	967	-1266
**Deaths**	**Effect size[a]**	**Denominator**	**Observed**	**Counterfactual**	**Deaths averted**
Child U5	*0.74*	26173	438	592	154
Child U5 pneumonia	*0.52*	4233	157	302	145

Calculated over 15-month post-intervention period (Apr 2021 to Jun 2022) by applying the effect size to the relevant population, then calculating the difference from what was observed.

a) effect size for children and children with pneumonia from Lam, F., et al. (2021). "Oxygen systems strengthening as an intervention to prevent childhood deaths due to pneumonia in low-resource settings: systematic review, meta-analysis and cost-effectiveness." BMJ Glob Health **6**(12).

Table S7 Exploratory analysis of subpopulations and their likelihood of receiving oxygen and odds of death (restricted to post-intervention period Jul 2021 to Jun 2022)

	Overall			Neonates						Children					
							Odds of death		OR no				Odds of death		OR no
**Population**	**N**	**O_2**	**% O_2**	**N**	**O_2**	**% O_2**	**O_2**	**No O_2**	**O_2 vs O_2**	**N**	**O_2**	**% O_2**	**O_2**	**No O_2**	**O_2 vs O_2**
SpO2 <90%	4077	3020	74.07%	2469	1901	76.99%	0.251534	0.220982	0.878539	1608	1119	69.59%	0.146417	0.08046	0.549523
SpO2 <90% + Hypox signs	1608	1362	84.70%	1317	1112	84.43%	0.286396	0.298013	1.040563	291	250	85.91%	0.152074	0.142857	0.939394
SpO2 <90% no Hypox signs	2469	1658	67.15%	1152	789	68.49%	0.205087	0.181818	0.88654	1317	869	65.98%	0.144772	0.075	0.518056
SpO2 90-93% + Hypox signs	337	179	53.12%	243	141	58.02%	0.169492	0.064516	0.380645	94	38	40.43%	0.117647	0.018868	0.160377
SpO2 90-93% no Hypox signs	2988	376	12.58%	1058	175	16.54%	0.082278	0.030562	0.37145	1930	201	10.41%	0.105556	0.017694	0.167625
SpO2 94% + Hypox signs	829	210	25.33%	434	183	42.17%	0.153846	0.06278	0.408072	395	27	6.84%	0.08	0.022989	0.287356
SpO2 94% no Hypox signs	27083	496	1.83%	4498	255	5.67%	0.09607	0.022727	0.23657	22585	241	1.07%	0.091743	0.00716	0.078045
Missing SpO2 + Hypox signs	163	61	37.42%	115	59	51.30%	0.647059	0.125	0.193182	48	2	4.17%	1	0.046512	0.046512
Missing SpO2 no Hypox signs	3583	67	1.87%	513	42	8.19%	0.333333	0.038462	0.115385	3070	25	0.81%	0.1	0.007543	0.075431
Missing SpO2	3746	128	3.42%	628	101	16.08%	0.5	0.047414	0.094828	3118	27	0.87%	0.047619	0.008843	0.185709

Table S8 Exploratory analysis of those with severe hypoxaemia (SpO2<90%) who do not receive oxygen on admission (restricted to post-intervention period Jul 2021 to Jun 2022)

	Overall (N=1057)		Neonates (N=568)		Children (N=489)	
**Population**	N	%	N	%	N	%
Signs of hypoxaemia (respiratory distress, chest indrawing, cyanosis, grunting, nasal flaring, drowsy, unconscious)	246	23.3%	205	36.1%	41	8.4%
WHO Emergency signs (altered consciousness, severe respiratory distress, uanable to feed/drink, chestindrawing in older child)	531	50.2%	352	62.0%	179	36.6%
In hospital death	134	12.7%	99	17.4%	35	7.2%
Received oxygen after admission	534	50.5%	360	63.4%	174	35.6%
Oxygen on day 2	134	12.7%	86	15.1%	48	9.8%
Oxygen on day 3	35	3.3%	20	3.5%	15	3.1%
SpO_2<90% on day 2	25	2.4%	17	3.0%	8	1.6%
SpO_2<90% on day 3	6	0.6%	5	0.9%	1	0.2%

Figure S3 Pulse oximetry coverage (proportion of admissions with documented SpO_2) over time, by age group and facility type

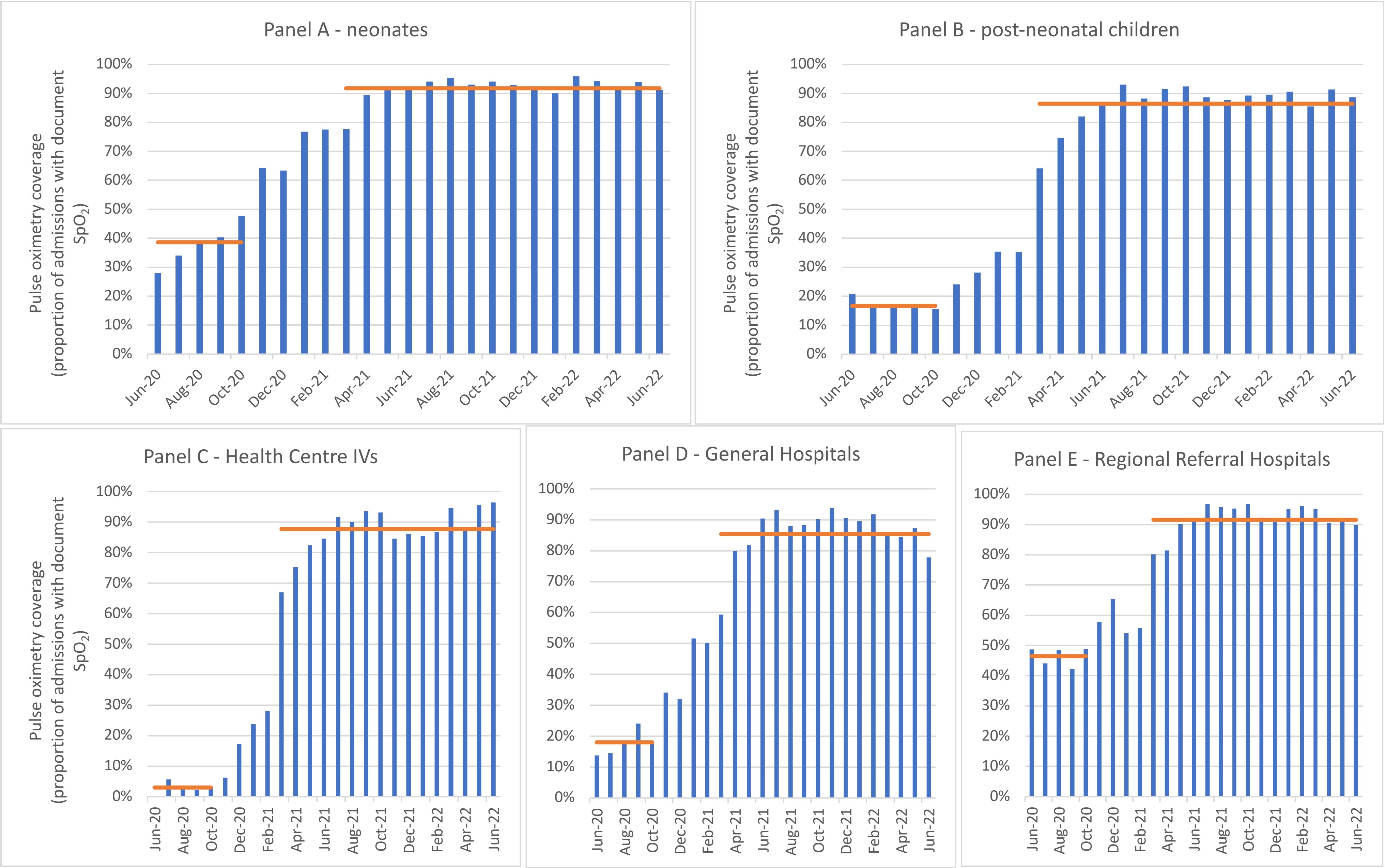

Figure S4 Oxygen coverage to hypoxaemic patients (SpO_2<90% or clinical signs of hypoxaemia if no pulse oximetry), by age group and facility type

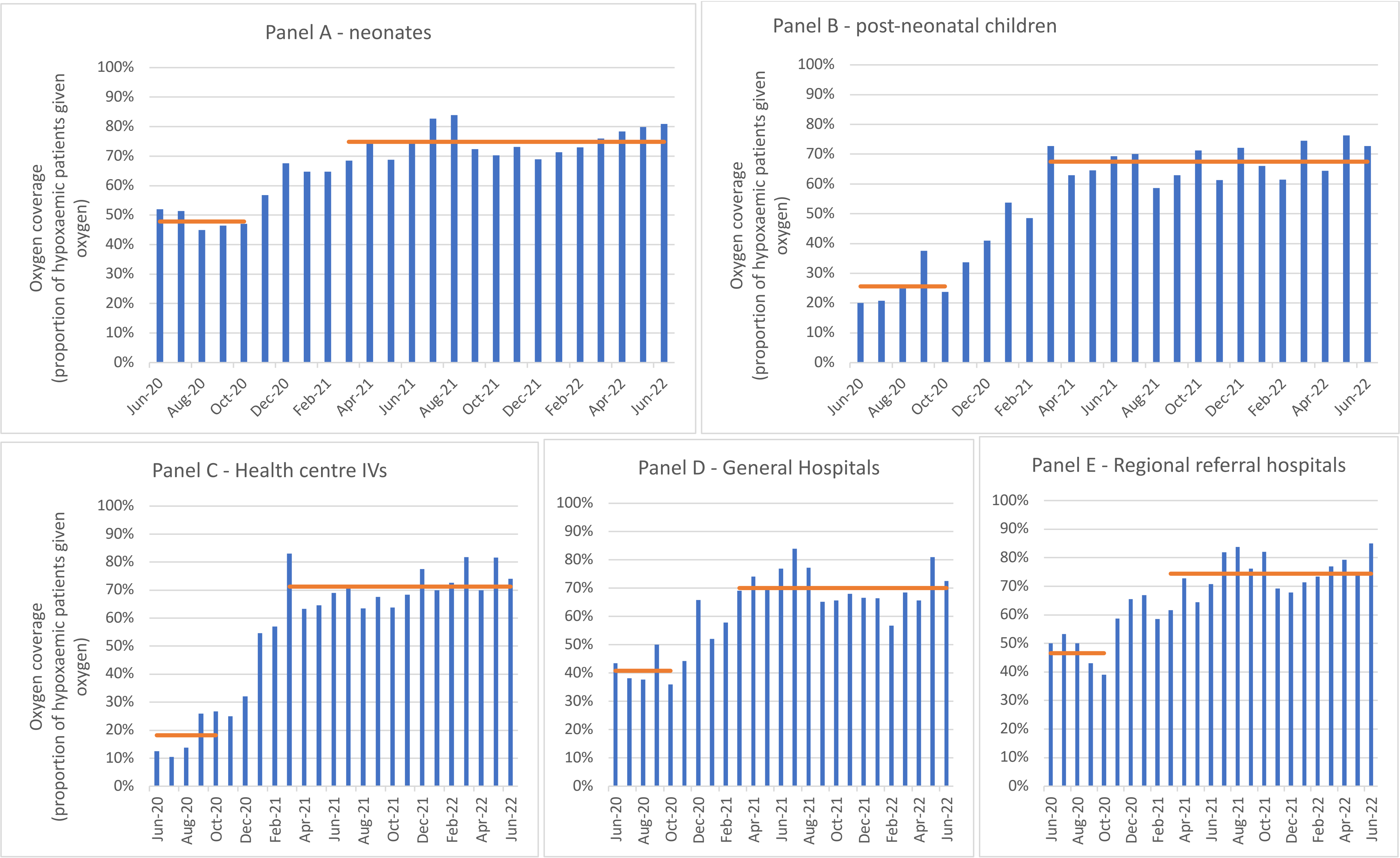
Panel A - neonates
Panel B - post-neonatal children
Panel C - Health centre IVs
Panel D - General Hospitals
Panel E - Regional referral hospitals
Oxygen coverage
(proportion of hypoxaemic patients given oxygen)
100%
90%
80%
70%
60%
50%
40%
30%
20%
10%
0%
Jun-20
Aug-20
Oct-20
Dec-20
Feb-21
Apr-21
Jun-21
Aug-21
Oct-21
Dec-21
Feb-22
Apr-22
Jun-22

Figure S5 Oxygen usage to all patients, by age group and facility type

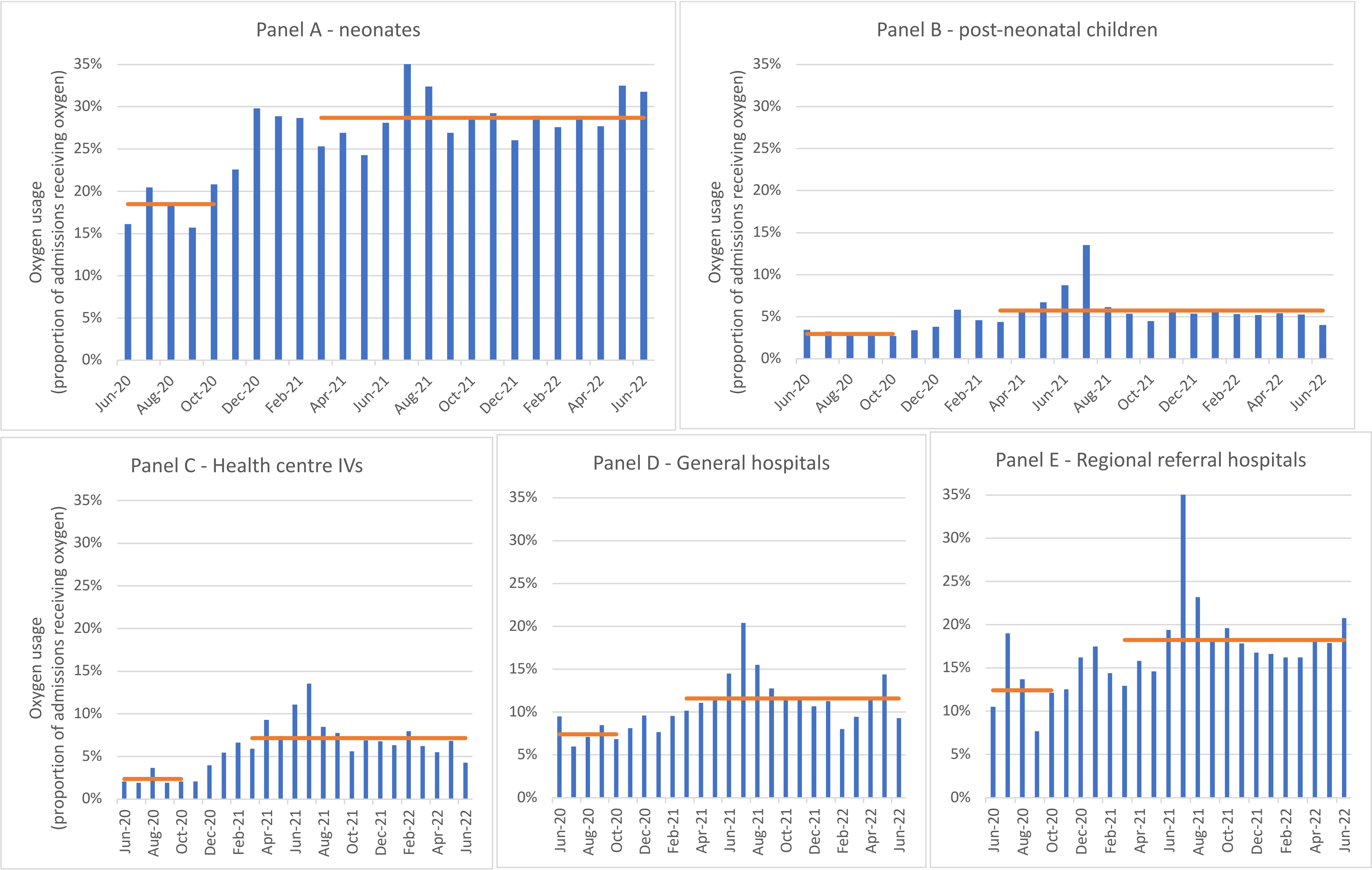

Supplement: Supplementary appendix 3 [file mmc3.pdf]
