# Supplementary material for: Improving effective coverage of medical-oxygen services for neonates and children in health facilities in Uganda: a before–after intervention study
Source: Lancet Glob Health. 2024 Aug 14;12(9):e1506–16. doi: 10.1016/S2214-109X(24)00268-7 (PMC11345447; doi:10.1016/S2214-109X(24)00268-7)
Supplement: Equitable Partnership Declaration [file mmc4.pdf]

THE LANCET
Global Health

Supplementary appendix 4

This Equitable Partnership Declaration (EPD) was submitted by the authors, and we reproduce it as supplied. It has not been peer reviewed. *The Lancet*'s editorial processes have not been applied to the EPD.

Supplement to: Graham HR, Kitutu FE, Kamuntu Y, et al. Improving effective coverage of medical-oxygen services for neonates and children in health facilities in Uganda: a before–after intervention study. *Lancet Glob Health* 2024; **12:** e1506–16.

Equitable Partnership Declaration questions

Researcher considerations

1. Please detail the involvement that researchers who are based in the region(s) of study had during a) study design; b) clinical study processes, such as processing blood samples, prescribing medication, or patient recruitment; c) data interpretation; and d) manuscript preparation, commenting on all aspects. If they were not involved in any of these aspects, please explain why.

   This question is intended for international partnerships; if all your authors are based in the area of study, this question is not applicable.

   This should include a thorough description of their leadership role(s) in the study. Are local researchers named in the author list or the acknowledgements, or are they not mentioned at all (and, if not, why)? Please also describe the involvement of early career researchers based in the location of the study. Some of this information might be repeated from the Contributors section in the manuscript. Note: we adhere to ICMJE authorship criteria when deciding who should be named on a paper.

a) Study design:

The study authors include researchers based in Uganda, the setting of the study; namely, FEK, YK, BK, SE, AZ, DK, LNK, CO, HA and FS and international collaborators (HRG, JM, FL). HRG, FL, JM, YK, BK conceived of the study design and prepared the first draft of the research protocol. FEK, YK, BK, SE, DK, LNK, CO led the submission to local ethics review, approval, registration and administrative clearance. All the co-authors addressed the comments raised by the ethics committee. YK, BK, SE, FEK, LNK led data collection and implementation. YK, BK, SE, LNK, AZ, DK, HA participated in the implementation of the different components of the multi-faceted intervention. BK, SE, JM and FL conducted data quality checks including field visits to the study health facilities. CO provided ongoing guidance on policy questions, adaptation of the intervention components to the context.

This study was conducted in the context of a long-standing collaboration between the Uganda Ministry of Health, Clinton Health Access Initiative (CHAI) and Makerere University joined by Murdoch Children's Research Institute (MCRI)/University of Melbourne, Australia as academic partners, respectively. Clinton Health Access Initiative has supported the Uganda Ministry of Health of Health since 2018 on efforts to strengthen the national medical oxygen ecosystem. FEK with mentorship of FS of Health Systems and Management at Makerere University formally joined the collaboration in 2020. At the time, CHAI was working with MCRI on the work in Uganda and other countries. At the Ministry of Health, CO, the Director of Health Services was a key collaborator and co-investigator on the study ensuring that the research questions were of importance to the Ministry of Health and providing additional context from the policy perspective. The team in Uganda was joined by HA from the Uganda Paediatric Association to provide expertise as an experienced paediatrician and additional context of the hypoxemia burden among the peadiatric population and challenges of access to pulse oximetry and oxygen therapy. The team of co-investigators led by CO (Uganda Ministry of Health), FEK and FS (Makerere University), HA (Uganda Peadiatric Association) and HRG (MCRI), a paediatrician and global leader on hypoxemia management and barriers of access to medical oxygen among those who need it, supported the CHAI team of YK, LNK, SE, DK, BK, AZ, JM, and FL to conduct the study. The academic partners led

the research component while the CHAI team led the implementation of the intervention that was evaluated.
**b) Clinical study processes:**<br>The health worker in-service training package and clinical mentorship package for health workers including biomedical engineers and technicians was developed with technical input from HRG, FEK, YK, SE, HA and CO. It was delivered by SE, AZ, DK and HA working with national team of training of trainers and regional oxygen champions. SE and HA joined the clinical mentorship in the study health facilities while YK, SE, AZ, DK and BK implemented the intervention components to provide catalytic medical oxygen equipment and devices and enhance the distribution and supply of medical oxygen. HRG, FEK, YK, BK, SE, JM, AZ, DK, HA, FL participated in the training of research assistants and pre-testing of the tools before initiating data collection. CO provided oversight and supervision of the implementation of the intervention components.
**c) Data interpretation:**<br>Data collection and management was led by BK and SE, assisted by DK and AZ on the data points about catalytic medical oxygen equipment and devices and the distribution and supply of medical oxygen to study health facilities. Data management and cleaning was conducted by BK, JM, FL under supervision of HRG. Data analysis was conducted by HRG, BK and FL. All the co-authors - HRG, FEK, YK, BK, SE, JM, AZ, DK, LNK, CO, HA, FS, FL – participated in multiple meetings of data exploration and interpretation.
**d) Manuscript preparation:**<br>The manuscript was drafted by HRG, with input from FEK, FL, JM, AB, YK, BK, SE, DK, LNK and AZ. FEK led the revisions to the manuscript. All authors contributed to revisions and approved the final manuscript. All authors (HRG, FEK, YK, BK, SE, JM, AZ, DK, LNK, CO, HA, FS, FL) had full access to all the data in the study and had final responsibility for the decision to submit for publication.

2. Were the data used in your study collected by authors named on the paper, or have they been extracted from a source such as a national survey? i.e., is this a secondary analysis of data that were not collected by the authors of this paper. If the authors of this paper were not involved in data collection, how were data interpreted with sufficient contextual knowledge?

   The Lancet Global Health *believe contextual understanding is crucial for informed data analysis and interpretation.*

A revised patient register form to capture summary data on SpO2 and oxygen therapy was introduced at the study health facilities. The health workers were trained about the revised patient register, which was to be integrated into the standard Health Management Information System reporting to MoH, subsequently. Research assistants trained by the study team used pre-tested data collection tools to extract individual patient data from case notes using a standardized electronic data collection form (SurveyCTO, Dobility Inc, Massachusetts, USA). They checked the ward registers to ensure inclusion of all eligible participants. Alongside the regular supportive supervision visits by clinical mentors and supervisors including BK, SE, HA and JM who are co-authors on the current paper. Additional support was provided by YK, LNK, FEK and CO who are based in Uganda and have sufficient contextual experience and knowledge.

3. How was funding used to remunerate and enhance the skills of researchers and institutions based in the area(s) of study? And how was funding used to improve research infrastructure in the area of study?

   Potentially effective investments into long-term skills and opportunities within institutions could include training or mentorship in analytical techniques and manuscript writing, opportunities to lead all or specific aspects of the study, financial remuneration rather than requiring volunteers, and other professional development and educational opportunities.

   Improvements to research infrastructure could be funding of extended trial designs (such as platform trials) and use of master protocols to enable these designs, establishment of long-term contracts for research staff, building research facilities, and local control of funding allocation.

**Skills:**<br>The CHAI team (YK, BK, SE, JM, AZ, DK, LNK, FL) worked closely on the different phases of study and intervention implementation with the researchers from the academic partners (HRG, FEK, FS) and acquired research skills including protocol design, research ethics considerations, data management and analysis, academic writing and preparation and delivery of dissemination products. BK, SE, JM, AZ, DK, LNK are junior researchers on the team and thus were supported by the investigators to understand the study and acquire skills to conduct the fieldwork, manage and participate in the analysis, interpretation and write up of the findings. They participated in preparation of the manuscript, and they are co-authors, in accordance with the ICMJE guidelines.
**Research infrastructure:**<br>This work has contributed to improving the research capacity at Makerere University, Uganda to conduct and generate evidence in the areas of implementation of proven and best practices in the quest to increase access to oxygen therapy solutions to reduce hypoxemia related morbidity and mortality. A research team lead by FEK and FS has emerged, and it closes collaborates with Ministry of Health, CHAI and other health sector partners to inquire into and generate evidence on implementation of oxygen therapy solutions to inform policy, programming and practice.

4. How did you safeguard the researchers who implemented the study?

   Please describe how you guaranteed safe working conditions for study staff, including provision of appropriate personal protective equipment, protection from violence, and prevention of overworking.

All the participating institutions (MoH, Mak, CHAI and MCRI) have policies on safe working conditions, equal employment opportunities, human resource management, off-site/field work conditions that were adhered to during the implementation of this study. These guidelines have provisions against any form of violence, overworking and exploitation. Field staff were provided with appropriate transport and appropriate personal protective equipment including face masks, alcohol rub during the COVID19 pandemic and other local disease outbreaks.

Benefits to the communities and regions of study

5. How does the study address the research and policy priorities of its location?

How were the local priorities determined and then used to inform the research question? Who decided which priorities to take forward? Which elements of the study address those priorities?

Pulse oximetry and oxygen therapy are life saving innovations needed in the management of several acute, chronic and surgical conditions across the life course in different healthcare settings. The coverage of pulse oximetry and oxygen therapy has remained low disproportionately affecting low-income countries like Uganda more than middle- and high-income countries, and it has largely neglected and affected by long standing under-investment in health systems. As such, many patient populations do not receive the health dividend promised by these proven innovations and die from preventable and treatable hypoxemia-related complications. In 2018, the Uganda Ministry of Health promulgated the first ever medical oxygen scale up implementation plan. There was thus a need to test and examine interventions from an implementation perspective to understand how to scale up pulse oximetry and oxygen therapy at different levels of the health system and what co-interventions are feasible and effective. This study therefore was conceptualized with this background in mind to generate evidence to inform policy, programming and practice and strengthen some existing policies.

6. How will research products be shared in the community of study?

For instance, will you be providing written or oral layperson summaries for non-academic information sharing? Will study data be made available to institutions in the region(s) of study? The Lancet Global Health *encourages authors to translate the summary (abstract) into relevant languages after paper editing; do you intend to translate your summary?*

Findings from this study have been disseminated at national level to all the key stakeholders including the Uganda Ministry of Health and representatives from the study health facilities. This paper will be published as open access. Once successfully published, it will be distributed shared through existing platforms in Uganda where the policy makers and health workers will have access to it. Lay person summaries in the local languages where the study was conducted have been prepared for non-academic information sharing.
We will use this to underpin discussions with the different policy makers and stakeholders at health facility, district and national level in Uganda. The findings from the study have been used to inform practice and strengthen existing policies and guidelines such as implementation of hypoxemia management and oxygen therapy guidelines, the essential medicines and health supplies list, the national quantification and forecasting of the medical oxygen need and medical oxygen supply chain.

7. How were individuals, communities, and environments protected from harm?

a) How did you ensure that sensitive patient data was handled safely and respectfully? Was there any potential for stigma or discrimination against participants arising from any of the procedures or outcomes of the study?

We have anonymised the participants in the text, and study obtained waiver of informed consent to collect anonymised data from the patient registers. Administrative clearance was obtained from the Ministry of Health and participating health facilities.

b) *Might any of the tests be experienced as invasive or culturally insensitive?*

Not applicable

c) *How did you determine that work was sensitive to traditions, restrictions, and considerations of all cultural and religious groups in the study population?*

Not applicable.
The interventions are part of what is approved as best practices or standard of care.

d) *Were biowaste and radioactive waste disposed of in accordance with local laws?*

Not applicable

e) *Were any structures built that would have impacted members of the community or the environment (such as handwashing facilities in a public space)? If so, how did you ensure that you had appropriate community buy-in?*

Not applicable

f) *How might the study have impacted existing health-care resources (such as staff workloads, use of equipment that is typically employed elsewhere, or reallocation of public funds)?*

Not applicable.

8. Finally, please provide the title (eg, Dr/Prof, Mr/Mrs/Ms/Mx), name, and email address of an author who can be contacted about this statement. This can be the corresponding author.

Name: Freddy Eric Kitutu
Email: kitutufred@gmail.com
